# Clinical and metabolic consequences of a historic pathogenic lamin A/C founder variant

**DOI:** 10.1038/s41598-025-08495-0

**Published:** 2025-07-04

**Authors:** L. Y. Wong, T. Torfs, S. J. V. Vanherle, J. Janssen, G. R. F. Claes, S. L. V. M. Stroeks, M. M. A. Willemars, F. Schianchi, D. Kapsokalyvas, E. Weltjens, A. Swinnen, A. Strzelecka, I. P. C. Krapels, S. R. B. Heymans, J. Glatz, A. van den Wijngaard, H. G. Brunner, J. Broers, J. F. P. Luiken, M. F. Hoes, J. A. J. Verdonschot, M. Nabben

**Affiliations:** 1 Department of Clinical Genetics, Maastricht University Medical Center+, Maastricht, the Netherlands; 2 Department of Genetics and Cell Biology, Maastricht University, Maastricht, the Netherlands; 3 Department of Cardiology, Maastricht University, Maastricht, the Netherlands; 4 CARIM Cardiovascular Research Institute Maastricht, Maastricht University, Maastricht, the Netherlands; 5 European Reference Network for Rare, Low Prevalence and Complex Diseases of the Heart (ERN GUARD-Heart), Maastricht, the Netherlands; 6 Centre of Cardiovascular Research, Centre for Molecular and Vascular Biology, University of Leuven, Leuven, Belgium; 7 Department of Human Genetics, Radboud University Medical Center, Nijmegen, the Netherlands

**Keywords:** Experimental models of disease, Genetics research, Stem-cell research, Clinical trial design, Disease genetics, Pluripotent stem cells, Reprogramming, Cardiology

## Abstract

A novel *LMNA* p.(Glu105Leu) variant was identified in five families with dilated cardiomyopathy (DCM), revealed as a local founder variant originating approximately 650 years ago. Genetic testing and clinical analysis of 795 DCM patients demonstrated that probands with this variant typically present with severe DCM in their sixties, characterized by high prevalence of late gadolinium enhancement, arrhythmias, and conduction disorders. Time-to-event analysis suggested a later onset of clinical symptoms compared to other *LMNA* variants, with a trend towards longer event-free survival. Microscopic imaging of patient fibroblasts, induced pluripotent stem cell-derived cardiomyocytes (iPSC-CMs), and heart tissue confirmed structural nuclear *LMNA*-associated abnormalities. Patient iPSC-CMs exhibited distinct sarcomeric disorganization, increased glucose uptake and glycogen content, reduced mitochondrial function and biogenesis, and delayed contractile function. These findings support the pathogenicity of the variant and demonstrate its profound impact on structural and metabolic functions in cardiomyocytes.

**Supplementary Information:**

The online version contains supplementary material available at 10.1038/s41598-025-08495-0.

## Introduction

Dilated cardiomyopathy (DCM) is one of the leading causes of heart failure and heart transplantation globally^1,2^. The disease is defined by the presence of left ventricular dilatation and systolic dysfunction unexplained solely by abnormal loading conditions or coronary artery disease^3^. While the full genetic complexity of DCM is not yet fully understood, it is known that DCM can be caused by likely pathogenic variants in more than 15 genes, showcasing the heterogenous nature of DCM^4^. The *LMNA* gene is one of the most frequently mutated genes in familial DCM^5,6^.

The *LMNA* gene encodes for lamins A and C, which are major components of the nuclear lamina, a structure that lines the inner nuclear membrane^7^. Today, hundreds of likely pathogenic variants in the *LMNA* gene have been described, and have been associated with a spectrum of diseases, referred to as laminopathies, which affect muscle, heart, fat distribution and metabolic processes. The phenotype of patients with a laminopathy includes premature aging disorders, metabolic disturbances such as partial lipodystrophy, and myopathies, including DCM^8^. Metabolic abnormalities can manifest as insulin resistance and hypertriglyceridemia attributing an important role for lamins in regulating glucose and lipid metabolism^9,10^.

The disease course of *LMNA-*associated DCM is often severe, and is characterized by a high risk of sudden cardiac death. The disease expression varies among *LMNA* variant carriers, even within families. The exact mechanisms underlying the development of DCM in *LMNA* variant carriers remains unclear^5,6^.

Heart failure is characterized by profound changes in cardiac metabolism, including shifts in substrate utilization from fatty acids to glucose, impaired energy production due to mitochondrial dysfunction, and changes in metabolic pathways that significantly impact cardiac function^11–13^. However, the specific changes associated with *LMNA*-related DCM remain largely unexplored. Few studies using transgenic mouse models have provided insights into metabolic alterations and potential mitochondrial dysregulation in *LMNA*-related DCM^14–19^. For example, impaired oxidative phosphorylation and mitochondrial dysfunction have been demonstrated early in disease progression, even before the onset of symptoms^14–19^. Furthermore, *Lmna* knockout mice show a downregulation of genes involved in mitochondrial function and oxidative phosphorylation^19^.

In this study, we characterize a variant in *LMNA* p.(Glu105Leu), identified in six families from The Netherlands and Belgium. We have previously reported nuclear abnormalities in fibroblasts from carriers of *LMNA* p.(Glu105Leu)^20^. This variant provides an opportunity to elucidate specific metabolic alterations associated with *LMNA*-related DCM. By using induced pluripotent stem cell-derived cardiomyocytes (iPSC-CMs), we analyzed nuclear envelope structure, substrate metabolic changes, contractile function and the arrhythmic-features of this variant.

## Methods

This study was approved by the local institutional review board (METC-2021-0329). All methods were carried out in accordance with relevant guidelines and regulations. Blood samples were collected from 795 DCM patients diagnosed according to international standards following written informed consent from all subjects. We used blood samples of one patient to generate two independent iPSC lines (referred to as clone 1 and clone 2), a commercially available iPSC line was used as a control. The detailed methods section is available as Data Supplement.

### Written informed consent

Written informed consent was obtained from all participants prior to their enrolment in the study. Each participant was provided with a detailed explanation of the study’s objectives, procedures, potential risks, and benefits. They were given sufficient time to ask questions and were assured that their participation was voluntary and that they could withdraw at any time without any consequences. The consent forms were signed by the participants and a copy was provided to them for their records. This process ensured that all participants were fully informed and consented to the use of their blood samples for research purposes.

## Results

### Identification of a novel *LMNA* founder variant

Genetic testing in our cohort of 795 patients with DCM identified a (likely) pathogenic (P/LP) variant in *LMNA* in 25 patients (3.1%). One specific P/LP *LMNA* variant was identified in six unrelated probands: c.313_314delinsTT, p.(Glu105Leu). This variant was previously reported by our team^20^. It was not present in the Genome Aggregation Database (gnomAD, v4.1.0; https://gnomad.broadinstitute.org/) or the GoNL database (https://www.nlgenome.nl/menu/main/app-go-nl/ suggesting this could be a local founder variant.

Haplotype analysis was performed in 5 probands and 4 family members to investigate the origin of the variant. A shared haplotype of at least 5 STR markers was found covering a 4.62 Mb region surrounding *LMNA* in all probands, providing evidence for a common ancestry for these families (Supplemental Figs. 1 and 2). By calculating the age of origin of the variant (Supplemental Fig. 2), we found that it originated between 25.7 and 26.2 generations ago. Assuming 25 years per generation, the p.(Glu105Leu) variant originated between 643 and 656 years ago, around the 1400s. Through genealogical research, we were able to connect families A, B, C and E. The common ancestor of these four families lived ten generations ago in a small village at the border of the Netherlands and Germany. Family D was linked to the other families three generations above, although the precise nature of this connection remains uncertain. While circumstantial evidence suggests kinship likely exists, definitive genealogical evidence is still lacking (Supplemental Fig. 3).

### Clinical findings in patients with the *LMNA* p.(Glu105Leu) variant and family segregation

**Fig. 1:**
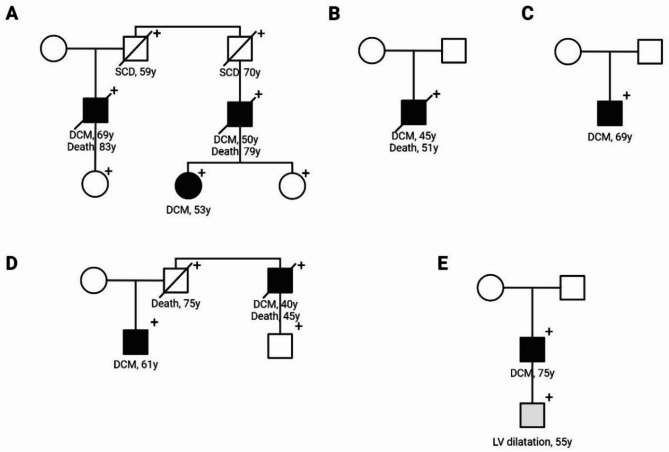
Pedigree of the families carrying the *LMNA* p.(Glu105Leu) variant. Squares represent males, circles represent females. Black symbol indicates an individual with a clinical diagnosis of dilated cardiomyopathy (DCM), a grey symbol indicates an individual with left ventricular dilatation, a crossed symbol indicates a deceased individual. ‘+’ indicates carriers of the variant.

The six probands with the *LMNA* p.(Glu105Leu) variant all presented with severe DCM. Ten additional family members with the *LMNA* variant were identified (Fig. 1). Five probands and 10 family members provided informed consent for further research (Table 1).

**Table 1:** Summary of baseline characteristics of the probands and family members carrying the *LMNA* p.(Glu105Leu) variant.

Characteristics	Probands (*n* = 5)	Family members (*n* = 10)
Age presentation/evaluation (years)	64 ± 12	60 ± 11
Male sex	5 (100)	7 (70)
NYHA class ≥ 3	2 (40)	0 (0)
Arrhythmias		
AV block (1st degree/total block)	1/1 (40)	1/0 (10)
Left bundle branch block	5 (100)	8 (80)
Atrial fibrillation	4 (80)	4 (40)
Non-sustained ventricular tachycardia	5 (100)	5 (50)
Percentage of VES per 24 h	28 [8–36]	2 [0–8]
Device implantation	4 (80)	1 (13)
Echocardiography*		
Left ventricular ejection fraction (%)	32 ± 7	56 ± 10
Left ventricular end-diastolic diameter (mm)	59 ± 4	53 ± 8
Indexed left atrial volume (ml/m^2^)	120 ± 43	75 ± 30
DCM diagnosis	5 (100)	3 (38)
Isolated left ventricular dilatation	0 (0)	1 (13)
MRI		
Left ventricular ejection fraction (%)	24 ± 3	-
Left ventricular end-diastolic volume (ml)	308 ± 122	-
Left ventricular end-systolic volume (ml)	237 ± 103	-
Stroke volume (ml)	71 ± 18	-
Late gadolinium enhancement (LGE)	4 (80)	-

Values represent mean ± standard deviation, absolute number (percentage) or median [interquartile range].

NYHA = New York Heart Association, AV = Atrioventricular, VES = Ventricular Extrasystoles, DCM = Dilated Cardiomyopathy, MRI = Magnetic Resonance Imaging.

* Data from echocardiography was available in 8 of the 10 family members.

The probands presented with DCM at a mean age of 64 ± 12 years, with an average LVEF of 32 ± 7% on echocardiography. The prevalence of arrhythmias and conduction disorders was 100%: all probands had a left bundle branch block and non-sustained ventricular tachycardias, while 80% also had atrial fibrillation. The median percentage of ventricular extrasystoles per 24 h was 28% [interquartile range 8–36%]. 80% of the probands had late gadolinium enhancement on the MRI. Although only 38% of the family members developed a DCM phenotype, already 80% had a left bundle branch block, 40% had atrial fibrillation and 50% had non-sustained ventricular tachycardias. This indicates that electric disturbances were already present before signs of systolic dysfunction were observed. None of the family members had a MRI.

### Time-to-event analysis demonstrates an age-dependent penetrance of *LMNA* p.(Glu105Leu) and longer event-free survival

We compared the age-dependent penetrance and event-free survival (composite endpoint of heart failure hospitalization, cardiac death, or left ventricular assist device implantation) between individuals with the p.(Glu105Leu) variant in *LMNA* and individuals with a different P/LP variant in *LMNA.*

**Fig. 2:**
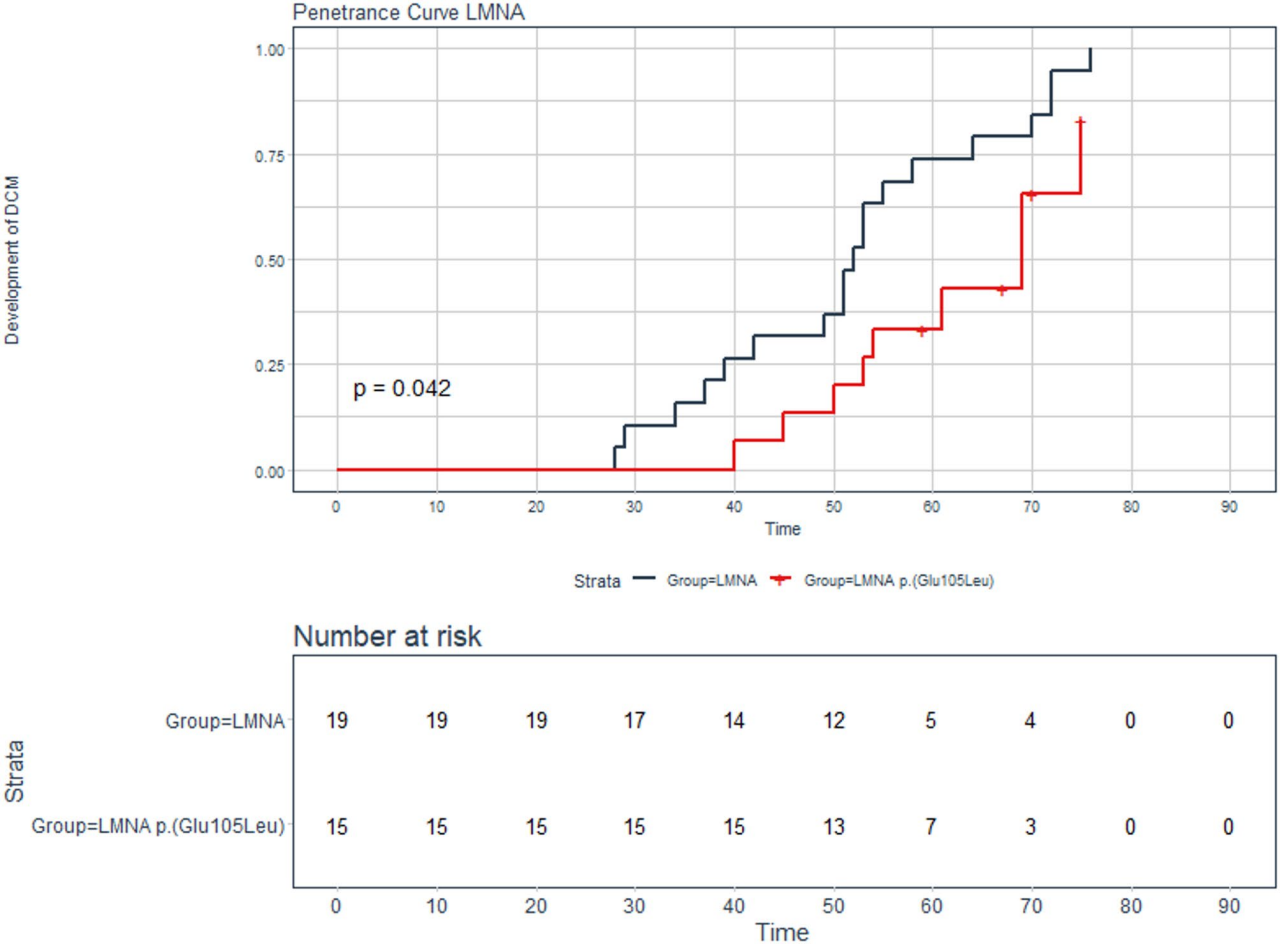
Age-dependent disease penetrance of *LMNA* variants. Kaplan-Meier curve showing the age-dependent penetrance of clinical symptoms in patients with the *LMNA* p.(Glu105Leu) variant (red) compared with a group of individuals with other P/LP *LMNA* variants (black). The time reflects the biological age of the individuals.

**Fig. 3:**
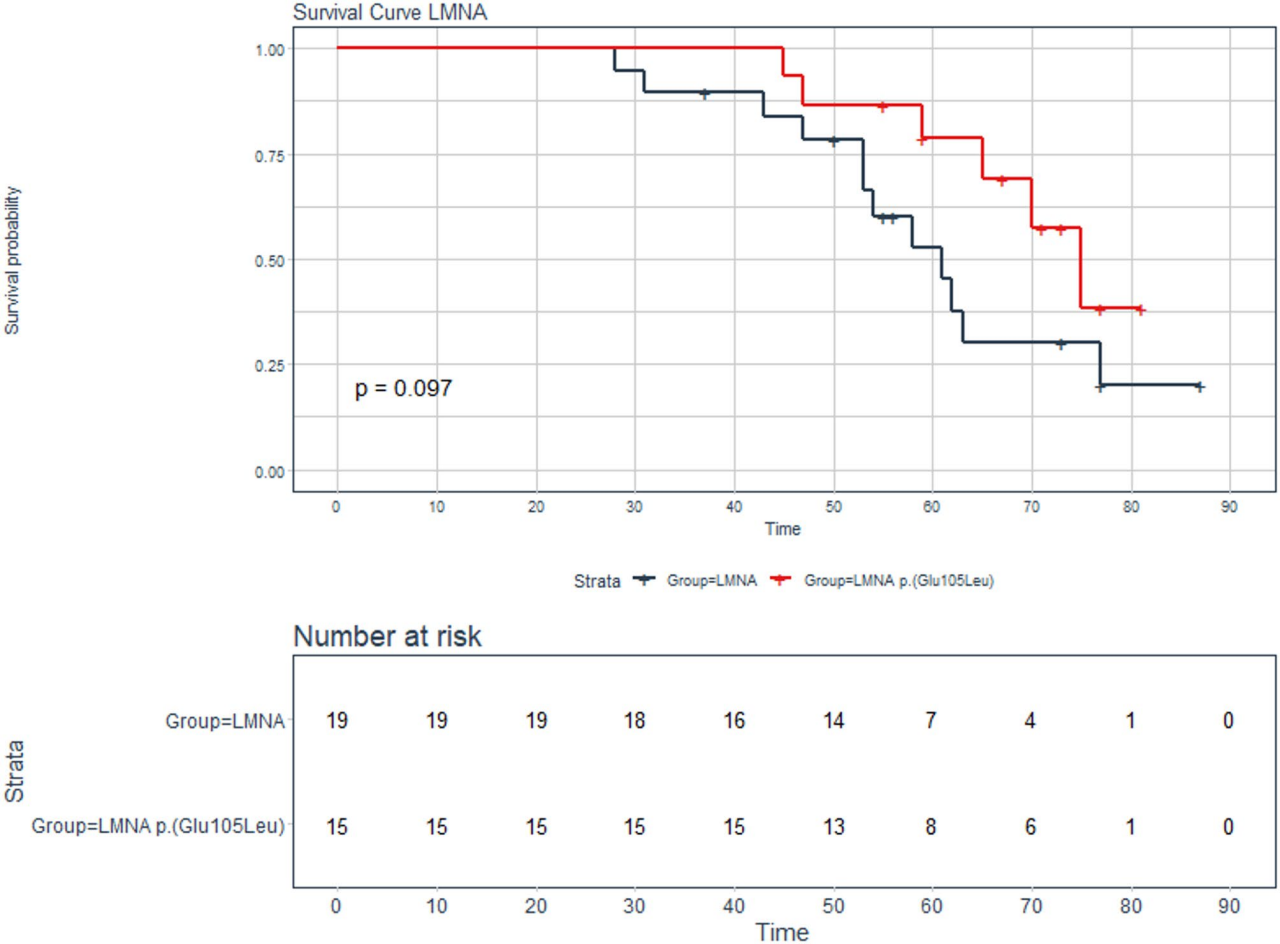
Event-free survival analysis of *LMNA* variant carriers. Kaplan-Meier curve showing the event-free survival (heart failure hospitalization, cardiac death, or left-ventricular assist device implantation) in patients with the *LMNA* p.(Glu105Leu) variant (red) compared with a group of individuals with other P/LP *LMNA* variants (black). The time reflects the biological age of the individuals.

The age of onset of clinical presentation in patients with the p.(Glu105Leu) variant occurs at a significant later age compared to other P/LP variants in *LMNA* (*p* = 0.042, Fig. 2). Seven out of 15 individuals with the *LMNA* p.(Glu105Leu) variant reached the composite endpoint (47%), compared to 12 out of 19 individuals with a different P/LP variant in *LMNA* (63%). Although there is no significant difference, a trend of a longer event-free survival was observed for the *LMNA* p.(Glu105Leu) variant (*p* = 0.097, Fig. 3). The majority of other *LMNA* variants identified are missense variants, with one being a splice variant. A detailed overview of these variants is provided in Supplemental Table 1.

### Immunohistological staining shows prevalence of nuclear abnormalities in *LMNA* p.(Glu105Leu) fibroblasts and iPSC-derived cardiomyocytes

To investigate the molecular and functional effects of the *LMNA* p.(Glu105Leu) founder variant, patient PBMC were collected to generate two clonal iPSC lines (referred to as clone 1 and clone 2), and differentiated into iPSC-CMs. A commercially available iPSC line was used as a control cell line. All cell lines exhibited similar maturation statues, as detailed in the Supplemental section.

**Fig. 4:**
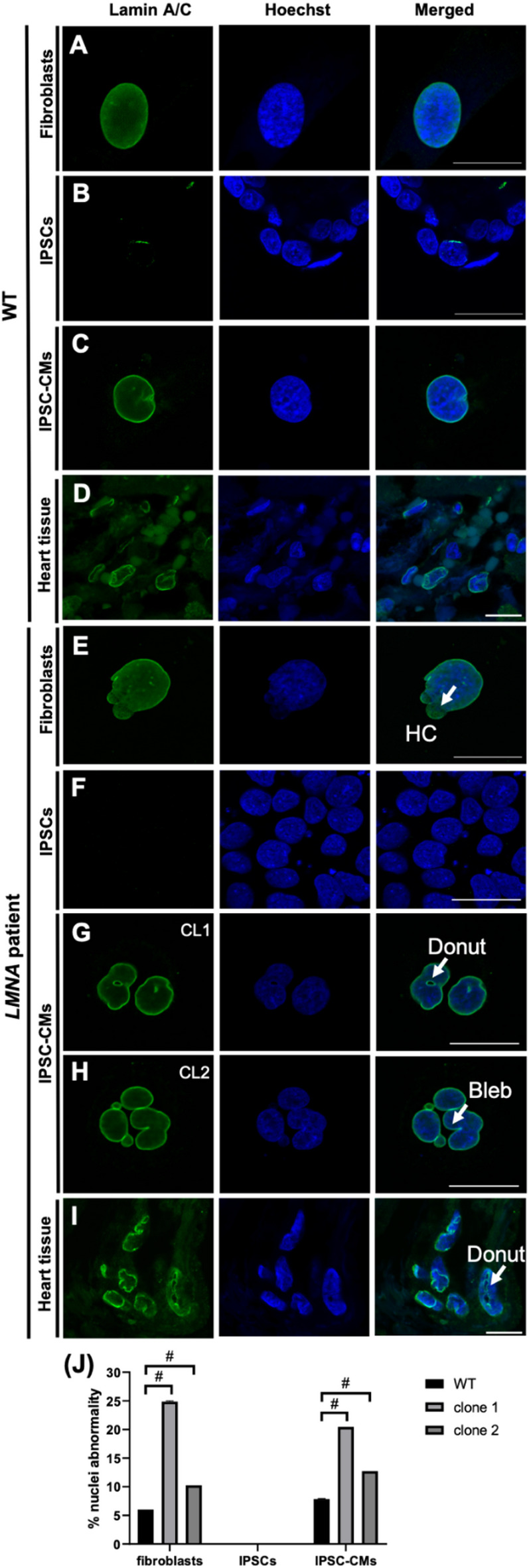
Nuclear envelope imaging of *LMNA* p.(Glu105Leu) and wild-type fibroblasts, iPSC-CMs and heart tissues. Immunohistological staining for lamin A/C (green) and Hoechst (blue) was used to assess the nuclear morphology in patient and wild-type fibroblasts obtained from unrelated controls, undifferentiated iPSCs, wild-type and patient iPSC-CMs, and heart tissue. Two independent iPSC lines (clone 1 and clone 2) were generated, along with one healthy unrelated control. The overlay images show the combined image of lamin A/C and Hoechst staining, allowing for the visualization of both the nuclear envelope and the nucleus (A-C, E-H) LMNA p.(Glu105Leu) fibroblasts, iPSC-CMs and heart tissue display a higher number of abnormal nuclei, as compared to the wild-type. In both LMNA p.(Glu105Leu) and wild-type undifferentiated iPSCs, no expression of Lamin A/C was observed, as expected. (D, I) Heart tissue from LMNA p.(Glu105Leu) patient shows similar abnormalities as those observed in the iPSC-CM model. (J) The percentage of nuclear abnormalities (including nuclear blebbing, donut structure and honeycomb structure), was significantly higher in LMNA p.(Glu105Leu) fibroblasts and iPSC-CMs compared to the wildtype. Scale bars: 20 μm (A-C; F-H); 10 μm (D and I). Data are represented as mean ± standard error of mean (SEM). Statistical significance was assessed using ANOVA and unpaired t-test. #*p* < 0.0001.

Immunohistological staining for lamin A/C and Hoechst was used to assess nuclear morphology in patient and wild-type fibroblasts obtained from controls, undifferentiated iPSCs, wild-type and patient iPSC-CMs and heart tissue. An irregular nuclear morphology, defined by honeycomb-like structures, blebbing and donut-shaped nuclei, was observed in the *LMNA* p.(Glu105Leu) fibroblasts (Fig. 4E–H), as compared to the control (Fig. 4A–D). In iPSC-CMs with the *LMNA* p.(Glu105Leu) variant, nuclear abnormalities were observed, with donut structures and nuclear blebbing being the most frequent observed irregularities. Similar abnormalities were observed in patient heart tissue (Fig. 4I), where donut structures in the nucleus were the most prevalent. Notably, in undifferentiated iPSCs derived from wild-type and patient cells, no nuclear abnormalities were detected (Fig. 4B, F). The morphology of 300 nuclei was assessed for both clone 1 and clone 2 (Fig. 4J).

The frequency of nuclear abnormalities observed in LMNA p.(Glu105Leu) iPSC-CMs is detailed in Table 2. In wild-type iPSC-CMs, 94.00% of nuclei were normal, with minimal abnormalities such as blebs (1.41%), donut-shaped nuclei (0.88%), and dysmorphic nuclei (1.94%). In contrast, clone 1 showed 74.70% normal nuclei, with increased frequencies of blebs (6.10%), donut-shaped nuclei (0.30%), and dysmorphic nuclei (5.49%). Clone 2 exhibited 71.36% normal nuclei, with notable abnormalities including blebs (5.34%), donut-shaped nuclei (1.21%), and dysmorphic nuclei (2.91%). Both clones also displayed various combinations of nuclear abnormalities, such as moon-shaped nuclei with blebs and honeycomb patterns.

**Table 2:** Frequency of nuclear abnormalities in *LMNA* p.(Glu105Leu) iPSC-CMs.

	Normal	B	Do	Dys	E	MN	MN + B	H	H + E	H + B	B + Dys	Do + Dys	B + Dys + MN	B + Dys + H	Moon	B + H + Moon
WT (%)	94,00	1,41	0,88	1,94	0,71	1,06	0,00	0,00	0,00	0,00	0,00	0,00	0,00	0,00	0,00	0,00
cl1 (%)	74,70	6,10	0,30	5,49	1,83	2,74	0,30	0,30	0,91	1,52	2,13	0,30	0,30	0,30	2,74	0,00
cl2 (%)	71,36	5,34	1,21	2,91	3,64	5,10	0,49	1,46	0,49	1,21	0,24	0,24	0,73	0,73	3,16	0,24

This table shows the frequency of nuclear abnormalities observed in *LMNA* p.(Glu105Leu) iPSC-CMs. The data includes the percentage of normal nuclei and nuclei with various abnormalities, such as blebs (B), donut-shaped nuclei (Do), dysmorphic nuclei (Dys), elongated nuclei (E), honeycomb-like nuclei (H), moon-shaped nuclei (MN), and combinations of these abnormalities. Three different cell lines are compared: wild-type (WT), cl1, and cl2.

Additionally, nuclear analyses of three patients’ fibroblasts in this family with identical variants, showed that the percentages of the different abnormalities were highly variable. For instance, in one patient honeycombs were rarely found, while in the two other patients honeycomb patterns were the most prominent abnormalities. The number of normal, Bleb (B) + micro, Honeycomb (H), Donuts (Do), B + HR, and B + Do nuclei in patients is shown in Table 3.

**Table 3:** Frequency of nuclear abnormalities in *LMNA* p.(Glu105Leu) patient fibroblasts.

	Normal	B + micro	H	Do	B + HR	B + Do
Family A; DCM, 53y (%)	89,8	10,0	0,0	0,3	0,0	0,0
Family B; DCM, 45y (%)	81,7	2,0	15,3	0,7	0,3	0,0
Family C; DCM, 69y (%)	76,3	6,3	9,3	7,0	0,7	0,3

The table displays the amount of normal, Bleb (B) + micro, Honeycomb (H), Donut (Do), B + H, and B + D nuclei in the patient of families A, B, and C.

### Ultrastructural imaging reveals structural disruptions, disorganized sarcomeres, altered mitochondrial distribution and increased glycogen content in *LMNA* p.(Glu105Leu) iPSC-derived cardiomyocytes

**Fig. 5:**
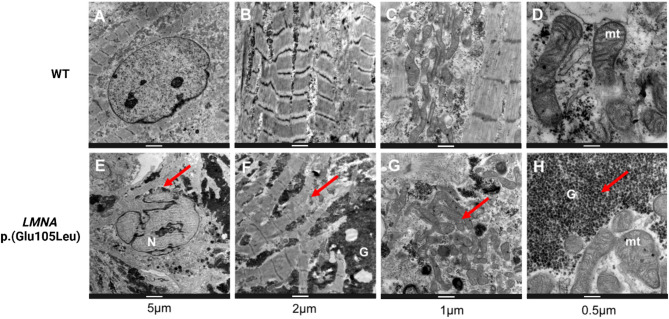
Transmission electron microscopy (TEM) imaging of wild-type (WT) and *LMNA* p.(Glu105Leu) iPSC-CMs. Wild-type iPSC-CMs show an (**A**) oval nucleus, (**B**) organized sarcomeric structures, (**C**) mitochondria aligned with the sarcomeres and (**D**) normal shaped mitochondria. In contrast, *LMNA* p.(Glu105Leu) iPSC-CMs show a (**E**) dysmorphic nucleus (**F**) disorganized sarcomeres surrounded with glycogen, (**G**) mitochondria clumped between sarcomeres and (**H**) mitochondria surrounded by glycogen. Annotation within figures: N = nucleus, mt = mitochondria, G = glycogen. Scale bars are indicated in each panel.

Transmission electron microscopy (TEM) allows detailed examination of cellular components. As illustrated in Fig. 5A-H, TEM analyses of patient-derived iPSC-CMs carrying the *LMNA* p.(Glu105Leu) variant revealed several structural alterations. First, a dysmorphic nucleus characterized by abnormal nuclear blebbing was confirmed in *LMNA* p.(Glu105Leu) iPSC-CMs (Fig. 5E), in agreement with the immunohistochemical data (Fig. 4). These abnormalities were not observed in wild-type iPSC-CMs.

Second, *LMNA* p.(Glu105Leu) iPSC-CMs exhibited scattered and disorganized sarcomeric structures (Fig. 5F). Additionally, the mitochondria in the patient iPSC-CMs were found to be clumped and interspersed among these sarcomeres (Fig. 5G), with extensive accumulation of glycogen surrounding the mitochondria and sarcomeres (Fig. 5H). Mitochondrial distribution differed between the wild-type iPSC-CMs and the *LMNA* p.(Glu105Leu) iPSC-CMs. The mitochondria in the wild-type iPSC-CMs were primarily located around the nucleus, while in the *LMNA* p.(Glu105Leu) iPSC-CMs, they were more dispersed throughout the cell.

While quantitative analyses of mitochondrial abnormalities and glycogen accumulation in *LMNA* p.(Glu105Leu) iPSC-CMs from TEM images (Fig. 5F-H) were not feasible, additional TEM images have been included in the Supplemental Results (Supplemental Fig. 8) to further illustrate these observations.

A Mitotracker staining was performed (Supplemental Fig. 6) to support the findings regarding mitochondrial distribution. In the wild-type iPSC-CMs the mitochondria are more located around the nucleus, whereas in the *LMNA* p.(Glu105Leu) iPSC-CMs, the mitochondria were more dispersed throughout the cell. Additionally, quantitative analysis of titin and $\:\alpha\:$-actinin IHC revealed an increase in sarcomeric dispersion and decreased aspect ratio of *LMNA* p.(Glu105Leu) iPSC-CMs, compared to the wild-type (Supplemental Fig. 7).

### *LMNA* p.(Glu105Leu) iPSC-CMs have an impaired bioenergetic profile

**Fig. 6:**
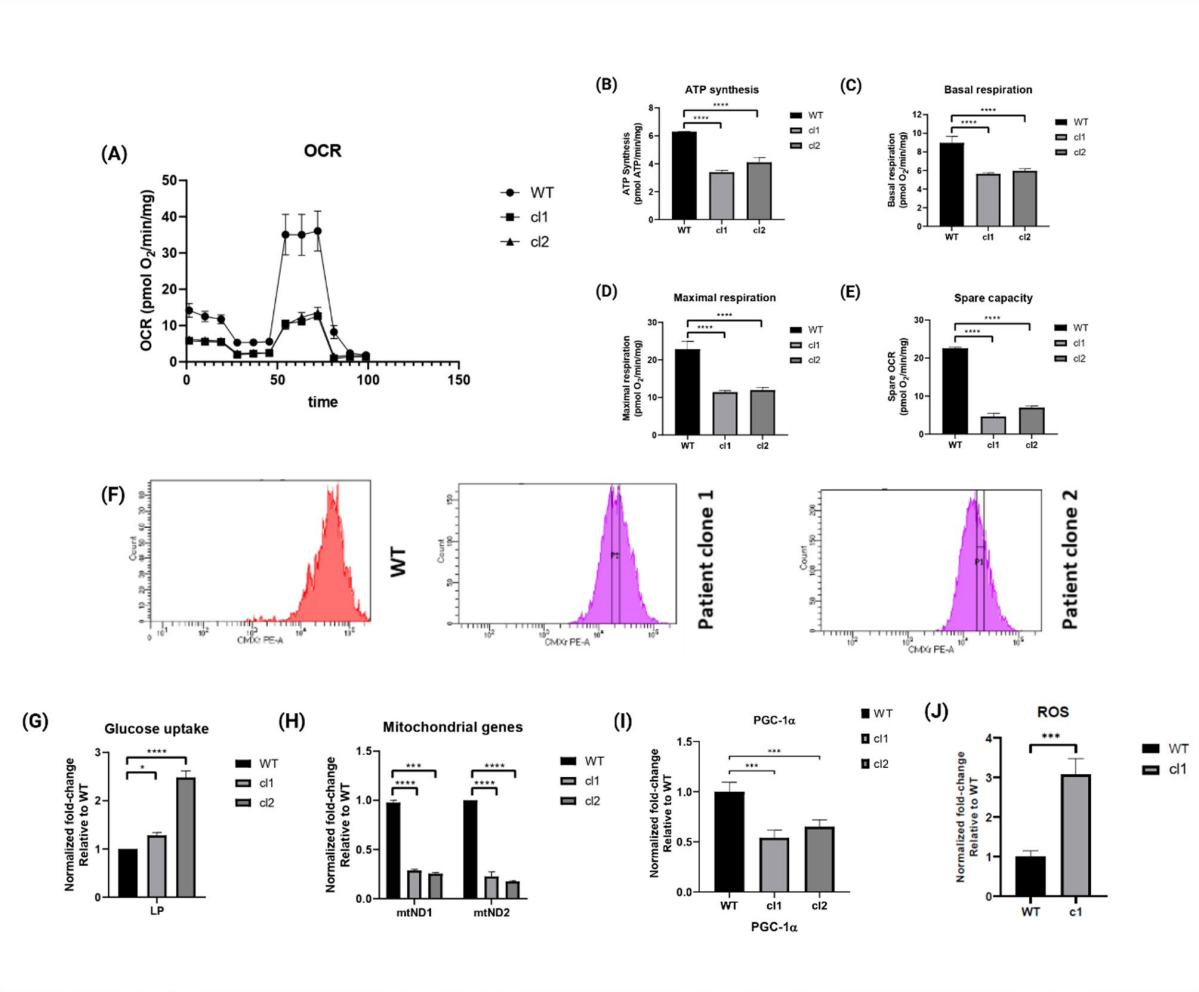
*LMNA* p.(Glu105Leu) iPSC-CMs have an altered bioenergetic profile. Two independent iPSC lines (clone 1 and clone 2) were generated, along with one healthy unrelated control, and subsequently differentiated into cardiomyocytes for these experiments. (**A**) Normalized oxygen consumption (OCR), the rates of (**B**) ATP synthesis, (**C**) basal respiration, (**D**) maximal respiration, and (**E**) spare capacity, were significantly lower in *LMNA* p.(Glu105Leu) iPSC-CMs, indicating an impaired metabolic activity in the patient iPSC-CMs. (**F**) Flow-cytometry analysis of mitochondrial content using Mitotracker CMXRed shows the distribution of mitochondrial mass in wildtype and *LMNA* p.(Glu105Leu) iPSC-CMs. (**G**) Glucose uptake assay results comparing iPSC-CM WT with iPSC-CM* LMNA* p.(Glu105Leu) clones 1 and 2 *LMNA* p.(Glu105Leu) iPSC-CMs show a 2.48 $\:\pm\:$ 0.46 fold increase in glucose uptake. Data are normalized to the total protein content, and the fold-change is relative to the condition ‘wildtype low palmitate’. (**H**) Decreased expression of mtND1 and mtND2, and (**I**) PGC-1α (assessed by qPCR), were substantially reduced in *LMNA* p.(Glu105Leu) iPSC-CMs, as compared to the wild-type. (**J**) An increase in ROS production was measured in *LMNA* p.(Glu105Leu) iPSC-CMs, as compared to the wild-type. Data were represented as mean ± SEM and plotted on Graphpad Prism (*n* = 3). Statistical significance was assessed using one-way ANOVA and unpaired t-test. *p < 0.05, **p < 0.01, ***p < 0.001.

In order to further explore the metabolic profile of the *LMNA* p.(Glu105Leu) variant, we analyzed glucose uptake in patient iPSC-CMs and the wild-type. Glucose uptake was found to be 2.5 $\:\pm\:$ 0.5 fold increased in *LMNA* p.(Glu105Leu) iPSC-CMs, as compared to the wild-type iPSC-CMs (Fig. 6G).

To assess whether the increased glucose uptake was parallelled by impaired mitochondrial respiration, a Seahorse XF assay was performed. This assay indicates that the *LMNA* p.(Glu105Leu) iPSC-CMs show impaired mitochondrial respiration. Patient clone 1 and clone 2 exhibit reduced basal and maximal oxidation consumption rate, reduced spare capacity and reduced ATP production rate as compared to the wild-type (Fig. 6A–E).

Flow cytometry analysis, using the MitoTracker CMXRos dye (see Supplemental Methods section for detailed description), showed a decreased number of mitochondria in *LMNA* p.(Glu105Leu) iPSC-CMs, as compared to the wild-type (Fig. 6F).

To confirm the observed decrease in mitochondrial mass, RT-qPCR was performed to assess the mitochondrial DNA expression by analyzing the expression of complex I-encoded genes mtND1 and mtND2. Additionally, the mRNA expression of PGC-1α, a key regulator of mitochondrial biogenesis, was evaluated. A lower *mtND1* and *mtND2* expression and a 2-fold lower PGC-1α expression was detected in the *LMNA* p.(Glu105Leu) iPSC-CMs, as compared to the wild-type (Fig. 6H–I).

Furthermore, we measured ROS production and found a 3.1 ± 0.4 fold increase in ROS in the *LMNA* p.(Glu105Leu) cells (Fig. 6J). This increase in ROS production is associated with a decrease in mitochondrial function.

To summarize, *LMNA* p.(Glu105Leu) exhibit a metabolic shift characterized by increased glucose uptake and glycogen content, alongside impaired mitochondrial respiration and reduced mitochondrial mass, compared to the control cell line.

### Impaired contractility and prolonged action potential duration in *LMNA* p.(Glu105Leu) iPSC-derived cardiomyocytes

**Fig. 7:**
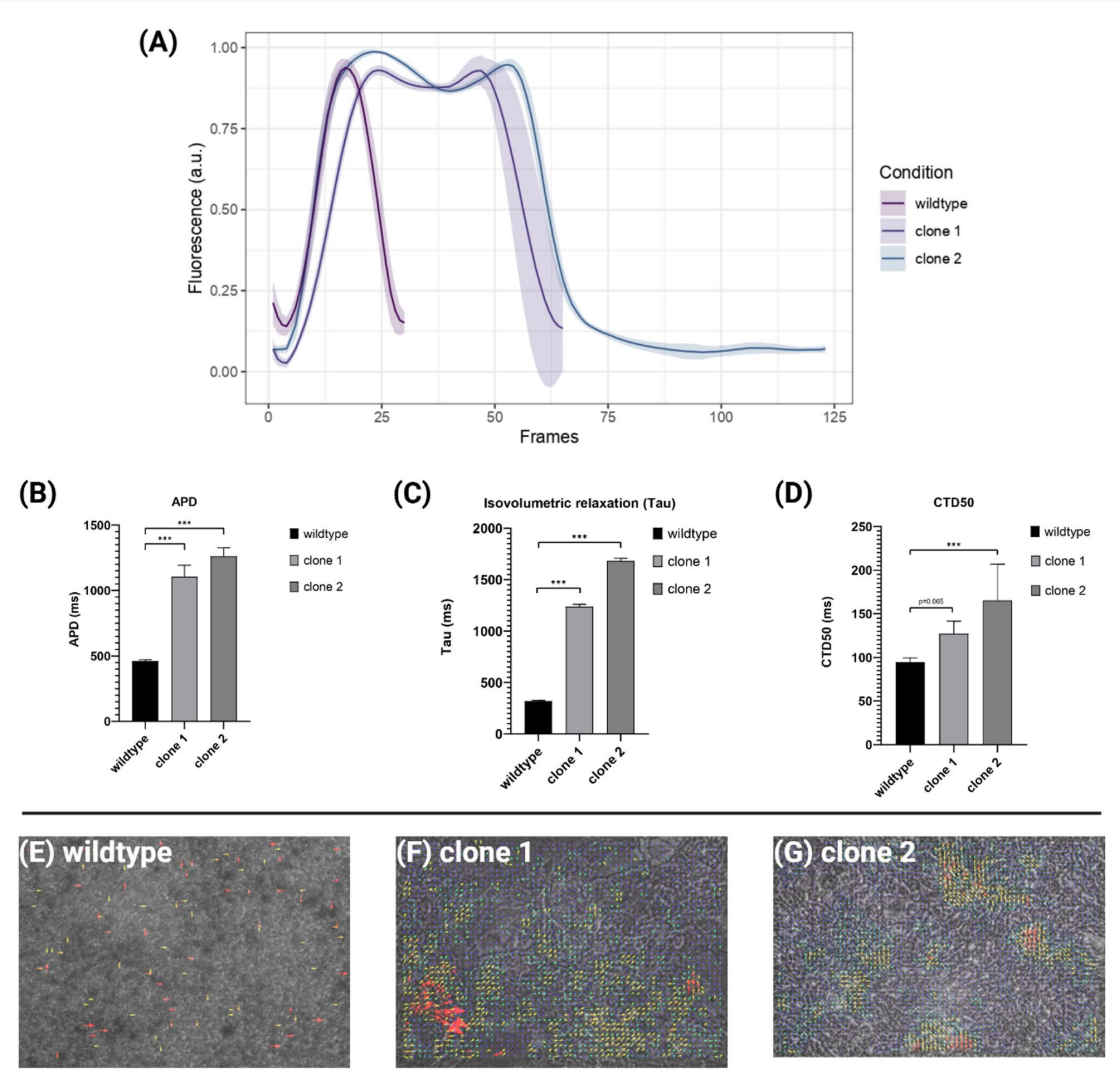
Analysis of contractile activity of both *LMNA* p.(Glu105Leu) iPSC-CMs clone 1 and clone 2 and wild-type iPSC-CMs. (**A**) Representative average contraction profiles. (**B**) Motion-based contractility analysis of iPSC-CMs reveals prolonged calcium transient duration, (**C**) prolonged relaxation time and (**D**) increased action potential duration (APD) in *LMNA* p.(Glu105Leu), as compared to the wild-type iPSC-CMs. (**E–G**) A particle image velocimetry (PIV)-determined map showing the spatiotemporal contraction of the iPSC-CMs. Data were represented as mean ± SEM and plotted on Graphpad Prism (*n* = 3). Statistical significance was assessed using one-way ANOVA and unpaired t-test. ***p < 0.001.

To assess cardiomyocyte contractility, we used action potential duration (APD), which measures the time for relaxation and contraction, and calcium transient duration (CTD), a marker linked to arrhythmogenic risk^21,22^. APD of the iPSC-CMs was measured by a video-based analysis, shown in Fig. 7. Under baseline conditions, *LMNA* p.(Glu105Leu) iPSC-CMs exhibited a decreased beating frequency as compared to their wild-type counterparts.

*LMNA* p.(Glu105Leu) iPSC-CMs also showed an increase in contraction duration and relaxation time, compared to the control. Additionally, a statistically significant prolonged CTD was observed for *LMNA* p.(Glu105Leu) iPSC-CMs compared to the wild-type. In conclusion, these findings demonstrate that the *LMNA* p.(Glu105Leu) variant alters the electrophysiological and contractile properties of iPSC-CMs, contributing to the pathogenesis of *LMNA*-associated cardiac disorders and indicating an increased risk of cardiac complications for individuals carrying this variant.

In addition to these measurements, we performed particle image velocimetry (PIV) analysis to compare the contractile behavior of the *LMNA* variant iPSC-CMs with the wild-type iPSC-CMs. The PIV analysis shows a difference in the contractile dynamics between the *LMNA* iPSC-CMs and the wildtype (Fig. 7E–G). Specifically, the *LMNA* p.(Glu105Leu) iPSC-CMs demonstrate a higher prevalence of warmer color spots in the overlay images, indicating increased contractile activity, compared to the wildtype. The increased contractility suggests that the *LMNA* variant may alter the mechanical properties of the cardiomyocytes. Notably, the PIV-results are in line with the increased APD and CTD, further supporting the evidence that the *LMNA* p.(Glu105Leu) variant increases the risk of cardiac complications.

In response to the prolongation of the APD in *LMNA* p.(Glu105Leu) iPSC-CMs, we investigated whether this electrophysiological phenotype was associated with altered expression of key genes involved in cardiomyocyte repolarization (Supplemental Fig. 4). Quantitative analysis of ion channel gene expression revealed an increase in *CACNA1C*, *KCNJ2*, *SCN5A*, and *RYR2* transcripts in the variant iPSC-CMs compared to the wild-type. Conversely, expression of *KCNQ1*, encoding a major component of the slow delayed rectifier potassium current (I_Ks), was decreased.

## Discussion

In this study we presented the clinical and functional characterization of a novel pathogenic founder variant *LMNA* p.(Glu105Leu), identified in six families with DCM. The clinical manifestation of the disease is characterized by a male predominance, high prevalence of LGE, conduction disorders and arrhythmias, and later onset of first symptoms compared to other *LMNA* variants. To prove the pathogenicity of this variant, we functionally characterized this variant using different cell types with the *LMNA* p.(Glu105Leu) variant in fibroblasts, patient heart tissue and iPSC-CMs showing structural, metabolic and functional abnormalities. In conclusion, this variant can be classified as pathogenic, with a phenotype consistent with *LMNA* variants, though milder in terms of significant clinical events and age of onset.

### The *LMNA* p.(Glu105Leu) variant is a founder variant and causes a late-onset DCM phenotype

A combination of haplotyping and genealogy determined that the p.(Glu105Leu) variant in *LMNA* is a local founder from the South of the Netherlands, creating an unique opportunity to investigate the variant-specific clinical and molecular consequences. The variant appears to have a later disease onset compared to other *LMNA* variants, with predominantly male probands presenting with DCM at a mean age of 64 ± 12 years. This later onset of disease suggests a potential milder phenotype, which is also supported by the trend towards longer event-free survival observed in our cohort compared with patients with other *LMNA* missense variants. While the later onset of this variant suggests a different phenotype, the occurrence of sudden death before the age of 50 in two families, indicates that the variant can still be associated with severe outcomes, thereby highlighting the importance of early recognition. A similar finding was observed for a different founder variant in *LMNA*, p.(Arg331Gln), which is also located in the α-helical rod domain of the protein^23^. It is already known that non-truncating variants in *LMNA* have a milder disease course compared to truncating variants^24^.

Our view on the clinical management of patients with DCM shifted from a disease-based approach towards a gene-specific approach, also apparent in the latest guidelines. Our data and others show that there is even clinical heterogeneity among specific variants within the same gene, potentially going towards an era of variant-specific management (such as in *PLN* p.(Arg14del)). Founder variants are a unique opportunity to investigate specific variants in more detail.

The high prevalence of arrhythmias and conduction disorders in family members with the p.(Glu105Leu) variant without signs of structural abnormalities or systolic dysfunction underscores the importance of close cardiac monitoring in all carriers of this variant. Notably, left bundle branch block and non-sustained ventricular tachycardia without systolic dysfunction were common, indicating significant electrical disturbances preceding systolic dysfunction. Therefore, it should be considered to screen all family members carrying this variant with at least echocardiography, ECG, and 24-hour ambulatory ECG. As the prevalence of LGE in patients with DCM and this variant was high, screening with MRI in family members should also be considered.

### Nuclear and ultrastructural abnormalities in *LMNA* p.(Glu105Leu) iPSC-CMs

The *LMNA* gene, which encodes for nuclear lamins A and C is expressed in well-differentiated cells and tissues. The absence of nuclear abnormalities observations in undifferentiated iPSCs is consistent with the known expression profile of the *LMNA* gene, as previous studies report that *LMNA* is only expressed in differentiated cell types^25–29^. Immunofluorescence and TEM imaging revealed structural abnormalities in patient-derived iPSC-CMs, fibroblasts, and heart tissue associated with the *LMNA* p.(Glu105Leu) variant. Nuclear analyses of three patients in this family with an identical variant showed that the percentage of different abnormalities were highly variable. However, the development of different types of nuclear abnormalities due to a specific variant is not yet fully understood. As we previously concluded, not the type of nuclear abnormality, but rather the determination of the percentage of abnormal cells can be used as a classifier for laminopathies^20^. The nuclear abnormalities observed in this study, including honeycomb-like structures, blebbing and donut-shaped nuclei, have also been examined in prior studies reporting laminopathies due to a variety of variants in *LMNA*^30,31^. These distinct nuclear morphologies are well-documented hallmarks of laminopathies, reflecting disruptions in nuclear envelope structure and function. Specifically, blebs or herniations indicate weakened lamina integrity, potentially due to disrupted lamin-chromatin interactions or mislocalization of lamin-binding proteins. Honeycomb structures suggest abnormal lamin polymerization patterns and chromatin disorganization, while donut-shaped nuclei may arise from defects in nuclear envelope reformation or altered connections between lamins and centrosomal proteins. Evidence from patient-derived cells, such as fibroblasts and cardiomyocytes, consistently demonstrates these nuclear phenotypes in association with *LMNA* variants^32^. These observations are consistent with the known role of lamins A/C in maintaining nuclear integrity. This is the first study reporting of consistent nuclear abnormalities in both heart biopsies, fibroblasts and patient-derived iPSC-CMs from patients carrying *LMNA* variants. Our findings align with the study by van Tienen et al., which also reported nuclear abnormalities in fibroblasts from carriers of *LMNA* p.(Glu105Leu)^20^.

Structural abnormalities of other cellular structures are relatively sparsely reported in laminopathies. The observed sarcomeric disorganization is consistent with earlier studies linking laminopathies to disturbed nucleo-cytoskeletal interactions^33^. Additionally, we observed clustering of mitochondria between the disorganized sarcomeres and glycogen accumulation which are indicative for underlying metabolic alterations.

It has been shown that nuclear shape abnormalities can lead to mechanical stress and excessive reactive oxygen species (ROS) production^34^. This mechanical stress and ROS production may contribute to the loss of mitochondria, further exacerbating the metabolic dysfunction observed in laminopathies. The disrupted mitochondrial distribution in *LMNA* p.(Glu105Leu) iPSC-CMs could be a downstream effect of the nuclear abnormalities. Our measurements revealed a 3.1 ± 0.4 fold increase in ROS in the *LMNA* p.(Glu105Leu) iPSC-CMs. This aligns with prior studies showing elevated ROS in laminopathy cell lines (e.g., R439C, R482W variants)^35^. This increase in ROS production is associated with a decrease in mitochondrial function and is thought to cause DNA damage in cardiomyocytes, leading to premature aging and prolonged decline in cardiac function^36,37^.

### Substrate metabolic alterations and mitochondrial dysfunction in *LMNA* p.(Glu105Leu) iPSC-CMs

Our findings suggest that the *LMNA* p.(Glu105Leu) variant significantly affects cellular organization and energy metabolism in cardiomyocytes, potentially contributing to laminopathy-associated cardiac dysfunction. Altered energy metabolism is often a key characteristic seen in heart failure. We therefore further investigated this in our *LMNA* variant. We observed increased glucose uptake in our *LMNA* iPSC-CMs, and altered electrophysiological and contractile properties.

While our findings suggest that metabolic abnormalities may play a role in the pathogenesis of *LMNA*-related DCM, it is important to acknowledge that the causal relationship between these metabolic changes and the development of DCM is complex. It is possible that the observed metabolic abnormalities are a consequence of changes in sarcomeric or contractile dynamics, rather than the cause. The nature of this relationship needs to be determined in further studies.

Mitochondrial changes included reduced mass, mtDNA copy number, and PGC-1α expression, indicating impaired biogenesis. This was accompanied by decreased oxidative phosphorylation and disrupted fusion-fission dynamics, further highlighting mitochondrial dysfunction. Comparable reductions in mitochondrial biogenesis have been reported in other laminopathies, such as Emery-Dreifuss Muscular Dystrophy (EDMD) and Hutchinson-Gilford Progeroid Syndrome (HGPS), with limited documentation in laminopathy-related DCM^38–41^. Hence, the increased glycogen content in iPSC-CMs, as seen with TEM, could be due to mitochondrial impairment and increased glucose uptake. This may contribute to the reduced contractility and pro-arrhythmic phenotypes. Increased glycogen content observed in our study aligns with findings from glycogen storage diseases, such as GSD III, where cardiac involvement is well-documented. Studies on GSD III have shown diffuse glycogen accumulation in cardiac structures, including the atrioventricular node and cardiomyocytes, leading to serious arrhythmias and heart failure^42^. This underscores the potential for glycogen accumulation to contribute to cardiac dysfunction, further supporting our observations in *LMNA*-related DCM.

The changes in expression of genes involved in cardiomyocyte repolarization suggest a remodeling of ion channel expression that may contribute to the prolonged repolarization phase and altered electrical properties observed in iPSC-CMs. Notably, we did not observe QT interval prolongation in patients despite reduced I_Ks, this could reflect compensatory interactions between these pathways. For instance, increased calcium/sodium currents may enhance depolarization, while elevated KCNJ2-currents could increase late repolarization. This may stabilize net repolarization duration, while preserving QT interval duration.

Our findings provide novel insights into *LMNA*-related DCM, linking structural, metabolic, and gene expression disruptions to the pathogenesis of this condition.

## Limitations and future outlook

This study provides valuable insights into the novel pathogenic *LMNA* p.(Glu105Leu) variant associated with DCM, as characterized in patient-derived iPSC-CMs. Despite certain limitations, we demonstrated significant metabolic alterations and identified nuclear envelope abnormalities and structural changes in the iPSC-CMs. Our findings align with the growing understanding that metabolic reprogramming plays a critical role in the pathogenesis of *LMNA*-related DCM.

However, there is one key limitation to address in future studies. Our sample size is relatively small, as we focused on two iPSC-CM clones derived from a single patient. While this allowed us to identify significant differences between *LMNA*-variant and control cells, a larger sample size would help confirm these results and explore potential mechanistic links across different *LMNA* variants.

Furthermore, we were not able to quantify the glycogen accumulation from the TEM images of our iPSC-CMs. The images were captures from different perspectives and made it challenging to obtain comparable data for quantification.

Looking forward, larger cohort studies, particularly those involving family members of the probands, will be crucial. This would provide additional insights into the disease progression and help refine screening recommendations for *LMNA*-related DCM. Additionally, expanding our analysis to include clinical MRI data from family members—especially examining whether late gadolinium enhancement (LGE) precedes conduction disorders—would be invaluable in understanding the disease’s trajectory and the potential utility of LGE as an early marker for cardiac involvement.

By addressing these limitations and expanding on the current findings, future research can provide a deeper understanding of *LMNA*-related DCM and its underlying pathogenic mechanisms, ultimately paving the way for targeted diagnostic and therapeutic approaches.

## Conclusion

In conclusion, this study characterizes the novel pathogenic *LMNA* founder variant p.(Glu105Leu) in iPSC-CMs in DCM. Clinical analysis revealed that probands presented with severe DCM in their sixties, with a high prevalence of LGE, arrhythmias and conduction disorders. Notably, even family members who had not yet developed DCM showed early signs of electrical disturbances. We have identified nuclear envelope abnormalities, structural changes and metabolic dysfunction in patient-derived iPSC-CMs. These findings suggest that metabolic alterations may play a role in the pathogenesis of *LMNA*-related DCM and underscore the importance of further research to explore potential therapeutic strategies.

## Electronic supplementary material

Below is the link to the electronic supplementary material.

Supplementary Material 1

## Data Availability

The datasets used and/or analyzed in this study are available from the corresponding author on reasonable request.
