# Supplementary material for: Clinical and metabolic consequences of a historic pathogenic lamin A/C founder variant
Source: Sci Rep. 2025 Jul 4;15:23842. doi: 10.1038/s41598-025-08495-0 (PMC12229498; doi:10.1038/s41598-025-08495-0)
Supplement: Supplementary file 1 — Supplementary Material 1 [file 41598_2025_8495_MOESM1_ESM.docx]

**SUPPLEMENTS**

**SUPPLEMENTAL METHODS**

**Subjects**

The study was conducted in accordance with the Declaration of Helsinki and received approval from the institutional medical ethics committee (METC-2021-0329). All human samples were obtained from patients with DCM and healthy controls following written informed consent with approval from the local institutional review board. Procedures were followed in accordance with institutional guidelines.

**WRITTEN INFORMED CONSENT**

Written informed consent was obtained from all participants prior to their enrolment in the study. Each participant was provided with a detailed explanation of the study's objectives, procedures, potential risks, and benefits. They were given sufficient time to ask questions and were assured that their participation was voluntary and that they could withdraw at any time without any consequences. The consent forms were signed by the participants and a copy was provided to them for their records. This process ensured that all participants were fully informed and consented to the use of their blood samples for research purposes.

**Fibroblast Cell Lines**

Skin fibroblast cultures were obtained from the Clinical Genetics (ClinGen) department of Maastricht UMC+ (MUMC+). Cells were derived from skin biopsies of two patients and a healthy individual.

Fibroblasts were grown in Dulbecco's modified Eagle medium (DMEM, Gibco, ThermoFisher Scientific) supplemented with 10 % (v/v) non-inactivated fetal bovine serum (FBS), 0.2 mM uridine (Acros), 1 % penicillin-streptomycin (Gibco, ThermoFisher Scientific). Cells were incubated at 37 ^o^C and 5 % CO_2_.

**Cardiomyocyte Maintenance and Differentiation**

Blood samples were obtained from patients and unrelated healthy controls. Pluripotent stem cells were derived, expanded and characterized by the RadboudUMC Stem Cell Technology Center (SCTC), <https://www.radboudumc.nl/en/research/radboud-technology-centers/stem-cells>, using peripheral blood mononuclear cells (PBMCs). A commercially available iPSC line was used as a control.

iPSCs were cultured in StemFlex medium (Thermofisher Scientific) on Matrigel-coated tissue culture plates and incubated at 37 ^o^C and 5 % CO_2_. Differentiation was initiated after the iPSCs reached 80 % confluence. On day 0 of differentiation, the culture medium was changed to RPMI 1640 GlutaMAX supplemented with B27-minus insulin (Gibco, ThermoFisher Scientific) containing CHIR99021 (Sigma Aldrich) and ascorbic acid (Sigma Aldrich). At day 2, the medium was changed to RPMI GlutaMAX-B27-minus insulin. After 24 hours, the medium was supplemented with Wnt-C59 (Cayman Chemical) and ascorbic acid. On day 5, the medium was changed to RPMI GlutaMAX-B27-plus insulin and maintained in this medium until the end of the differentiation.

Spontaneous beating cells were typically observed at day 10. Metabolic selection was started at day 14 with RPMI GlutaMAX-B27-minus glucose, supplemented with 5 mM sodium DL-lactate for four days.

**iPSC-CM Maturation**

After metabolic selection, iPSC-CMs were dissociated using 10X TrypLE, counted and replated at 3-4 x 10^6^ cells/well on 6-well plates for maturation in RPMI GlutaMAX-B27, supplemented with 10 % KOSR. After 2 days, the cells were cultured in RPMI-B27-minus glucose. At day 5 the cells were maintained in DMEM-minus glucose (Gibco, ThermoFisher Scientific), supplemented with 3 mM glucose (Sigma Aldrich), 10 mM L-lactate (Sigma Aldrich), 5 µg/mL Vitamin B12 (Sigma Aldrich), 0.82 µM Biotin (Sigma Aldrich), 5 mM creatine monohydrate (Sigma Aldrich), 2 mM Taurine (Sigma Aldrich), 2 mM L-carnitine (Sigma Aldrich), 0.5 mM Ascorbic acid (Sigma Aldrich), 1x NEAA (ThermoFisher Scientific), 0.5 % (w/v) Albumax (ThermoFisher Sientific), 1x B27 and 1 % KOSR (ThermoFisher Scientific).

To profile the maturation status of the iPSC-CMs the expression of several genes was assessed using qPCR including mature CM-related sarcomere protein encoding genes, (*TNNT2, TNNI3, MYH7*), ion channel genes (*SCN5A, KCNJ2*), and the genes of encoding calcium handling-related proteins (*RYR2, GJA3*) as shown in **Supplemental Figure 4**.

**PCR and Genome Sequencing**

DNA was extracted using the QIAmp DNA mini kit (Qiagen), according to the manufacturer’s recommendations and quantified using Nanodrop. PCR was performed in a volume of 20 µL containing 10 µM primers (IDT) and 1x Amplitaq Gold 360 (ThermoFisher Scientific) DNA polymerase. Sequencing was carried out with the ABI3703 Genetic Analyzer (Applied Biosystems).

**RT-qPCR Analysis**

RNA was extracted using the High Pure RNA isolation kit (Roche), according to the manufacturer’s recommendations. After RNA isolation 500 ng of total RNA was reverse-transcribed to cDNA using iScript Supermix (Quanta-Bio). RT-qPCR was performed with 2x Sensimix SYBR Hi-ROX (Biokine). cDNA was amplified on a Lightcycler 480 (Roche). cDNA was amplified in a volume of 10 µL, containing 2x Sensimix SYBR Hi-ROX (Bioline), 25 µmM primers (IDT) and 5x diluted cDNA template. Gene expression was analysed using the comparative $2^{-\Delta\Delta Cq}$ method and normalized to the internal reference.

**Ultrastructural Analysis (Transmission electron microscopy)**

Ultrastructural analysis on cultured iPSC-CMs was performed using transmission electron microscopy (TEM). In short, iPSC-CMs were fixed with 2.5 % glutaraldehyde and 1 % PFA in 0.1 M phosphate buffer containing 0.01 % CaCl_2_ and MgCl_2_. Cells were post-fixed in osmium tetraoxide and dehydrated using increasing ethanol concentrations (70 %,90 % and 100 %. Next, the cells were infiltrated and embedded with Epon. Ultrathin sections were prepared at 60 mm thickness with an ultramictocome (Leica UC7). Electron staining with uranyl acetate and lead citrate was applied. Images were captured with a Tecnai T12 Electron Microscope equipped with an Eagle 4kx4k CCD camera (ThermoFisher).

**Immunofluorescence Imaging**

Cells were fixed with 4 % paraformaldehyde (PFA) in PBS. After incubation in PBS, cells were permeabilized using 0.1 % Triton and non-specific binding sites were blocked with blocking buffer. The cells were immersed in Lamin A/C primary antibody (Santa Cruz, dilution 1:50) for 1 hour. After a washing step, the cells were stained with a secondary antibody (goat anti-mouse, Alexa Fluor 488, dilution 1:500) for 1 hour.

For the imaging of sarcomeric structures, the cells were incubated with primary antibodies, titin (9D10, Developmental Studies Hybridoma Bank, 5 µg/mL) and $\alpha$-actinin (A7811, Sigma Aldrich, 1:4000) for 45 minutes, after blocking. After a washing step, the cells were stained with secondary antibodies, Alexa Fluor goat anti-mouse 488 IgM, Alexa Fluor goas anti-mouse 568 IgG (Thermofisher Scientific, 1:500) and Hoechst for 1 hour. Images were analysed with Fiji.

**Seahorse XF Analysis**

Mitochondrial respiration was assessed using the Seahorse XF24 extracellular flux analyzer (Agilent Technologies). iPSC-CMs were seeded with a density of 40 x 10^5^ cells/well on a Matrigel coated XF24 Cell Culture Microplate, and were allowed to recover for three days. After three days of recovery, the cell culture medium was exchanged for Agilent XF RPMI Base Medium (Agilent Technologies) supplemented with 1 mM pyruvate (Agilent Technologies, 2 mM glutamine (Agilent Technologies) and 10 mM glucose (Agilent Technologies), 1 hour prior to the assay. The Mito Stress test was performed according to the manufacturer’s recommendations. The injected concentrations were as follows: oligomycin (2.0 µM, Sigma Aldrich), FCCP (1.0 µM, Sigma Aldrich), antimycin (0.5 µM, Sigma Aldrich) and rotenone (0.5 µM, Sigma Aldrich). The obtained data were normalized to protein content.

The baseline OCR was defined as the average values measured from time 1 to 3 during the experiments. Maximal OCR was the OCR difference between FCCP and antimycin, with reserve capacity being the difference between FCCP and baseline OCR values.

**Flow Cytometry**

iPSC-CMs were cultured in medium containing 250 nM of Mitotracker CMXRed (ThermoFisher Scientific) for 30 min, prior to dissociation. For flow cytometry, the cells were dissociated with TrypLE, washed with PBS, stained and fixed with 4 % paraformaldehyde. Post-fixation, the cells were permeabilized using 0.1 % Triton and aspecific binding sites were blocked with blocking buffer. Next, the cells were incubated with a primary antibody (cardiac troponin T, Abcam) for 1 hour. After a washing step, the cells were stained with a secondary antibody (goat anti-mouse, Alexa Fluor 488m ThermoFisher) for 1 hour. Flow cytometry was performed using BD FACS Aria (BD Biosciences), according to the manufacturer’s recommendations.

**Glucose Uptake Assay**

One day prior to the assay, 25 x 10^4^ iPSC-CMs were plated on a Matrigel-coated 24 well plate. Glucose uptake was evaluated after incubation with, or without, 100 nM insulin for 30 min. The uptake assays were performed as described previously by (Wang et al, 2024) ^37^.

**ROS Assay**

Reactive oxygen species (ROS) production was measured using the fluorescent probe 2′,7′-dichlorodihydrofluorescein diacetate at 10 μM in PBS for 30 min. Fluorescence was measured using the fluorescence SpectraMax iD3 reader at 495 nm excitation and 529 nm emission.

**Contractility Assay**

For contractility assessment, iPSC-CMs were dissociated and resuspended in cardiomyocyte replating medium, supplemented with KOSR and Rock inhibitor. Cells were plated at a density of 800 x 10^3^ cells/well in a Matrigel-coated 24 well plate, where they formed synchronous beating iPSC-CM monolayers. Prior to contractility measurements, the medium was changed to Tyrode’s solution (135 mM NaCl, 5 mM KCl, 1 mM MgCl_2_, 10 mM HEPES and 10 mM D-glucose). Contractile time series were recorded for 20 second intervals, using a Nikon high speed camera. Data were analyzed using MUSCLEMOTION software and PIV analyses on ImageJ. The analyzed metrics include CTD50, relaxation time and contraction duration. The assays were performed under baseline conditions and after supplementation of 0.5 µM isoproterenol.

**Statistical Analysis**

The experimental data are represented as the mean values with standard error of the mean (mean ± SEM). Statistical analysis was carried out using the student’s t-test, one-way ANOVA, two-way ANOVA, depending of the nature of the variables and whether the data met the assumptions of the specific tests. Statistical significance is defined at * (p < 0.05), ** (p < 0.01), *** (p < 0.001) and # (p < 0.0001). All the analyses were performed using GraphPad Prism.

**Genotyping and Haplotype Analysis**

Seven polymorphic microsatellite markers surrounding the *LMNA* gene were genotyped in all variant carriers from the families and three CEPH (Centre d’Etude du Polymorphisme Humain, France) individuals. The markers were chosen using the Marshfield Genetic maps (http://research.marshfieldclinic.org/genetics/GeneticResearch/compMaps.asp) and the UCSC genome browser (http://genome.ucsc.edu/). The markers are located in a region of 4.62 Mb with locus order CEN-D1S2346-D1S305-D1S2714-D1S2777-D1S2721-D1S2624-D1S1600-TEL on chromosome 1q21.3-23.1. Genotyping was performed on three individuals from the CEPH database to use the marker genotypes and allele frequencies provided in the CEPH genotype database. Patient allele sizes were extrapolated by comparing them to the existing allele sizes in the CEPH database. The frequencies of the disease-associated alleles were determined based on the data available in the CEPH database.

For PCR, a forward primer with an M13 tail, along with a universally fluorescent-labeled M13 primer was used. PCR products were analyzed on an ABI3730 automated sequencer utilizing the GeneMapper 4.0 software package.

**Calculation of Age of Founder variant**

The calculation of the age of origin of the *LMNA* p.(Glu105Leu) pathogenic founder variant in generations (G) was conducted as described by Risch, et al., as shown in Figure X. The distance between the closest recombinant microsatellite marker and the variant was used to measure the linkage disequilibrium ($\delta$), where $\delta$ = (Pd-Pn)/(1-Pn), Pd represents the frequency of the ancesteral allele among the chromosomes carrying the *LMNA* variant and Pn represents the frequency of the allele on the chromosomes without the mutated allele. CEPH allele frequencies were used to substitute Pn (http://www.cephb.fr/), and the USCS database (http://genome.ucsc.edu/) was used to determine genetic distances. The calculations are bases on the assumption that 1 Mb is approximately equivalent to 1 cM, where $\theta$ represents the recombination fraction (in Morgan, M) between the marker and the variant.

The linkage disequilibrium ($\delta$) was calculated for each of the closest recombinant microsatellite markers D1S2346 and D1S1600. For Pd, the genotypes from variant carriers from all subjects was used. Pd is 0.600 for D1S2346 and 0.800 for D1S1600, and $\delta$ = 0.460 for D1S2346 and $\delta$ = 0.643 for D1S1600. The genetic distances between the markers and the variant is approximately 0.0292 M for D1S2346 and 0.0170 for D1S1600.

**SUPPLEMENTAL TABLES**

**Supplemental Table 1 | Overview of all variants in *LMNA* in the control cohort (n=19)**

| **Specific *LMNA* variant (nucleotide and amino acid change)** | **Number of patients with the specific variant** |
| --- | --- |
| c.357-2A>G; p.(?) | 1 |
| c.1300G>A; p.(Ala434Thr) | 2 |
| c.236C>A; p.(Ala79Asp) | 2 |
| c.658C>T; p.(Arg220Cys) | 2 |
| c.992G>A; p.(Arg331Gln) | 2 |
| c.1130G>A; p.(Arg377His) | 1 |
| c.1303C>T; p.(Arg435Cys) | 1 |
| c.481G>A; p.(Glu161Lys) | 6 |
| c.1039G>A; p.(Glu347Lys) | 1 |
| c.810G>A; p.(Lys270Lys) | 1 |

**SUPPLEMENTAL FIGURES**

| 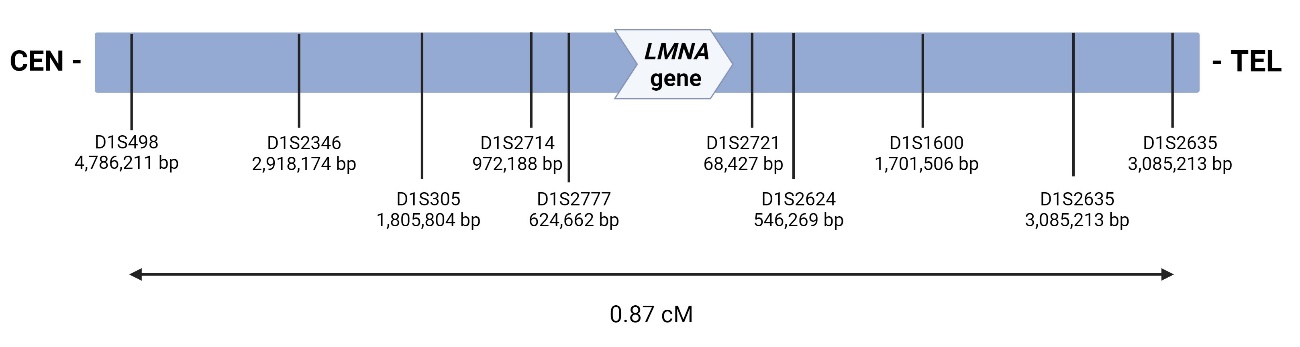 |
| --- |
| **Supplemental Figure 1. Schematic overview of the *LMNA* locus on chromosome 1**. The arrow indicates the locus with respect to chromosome orientation. Marker names and their physical distance (bp) to the *LMNA* locus are shown in the figure. Physical distances are derived from the USCS genome browser. |

| 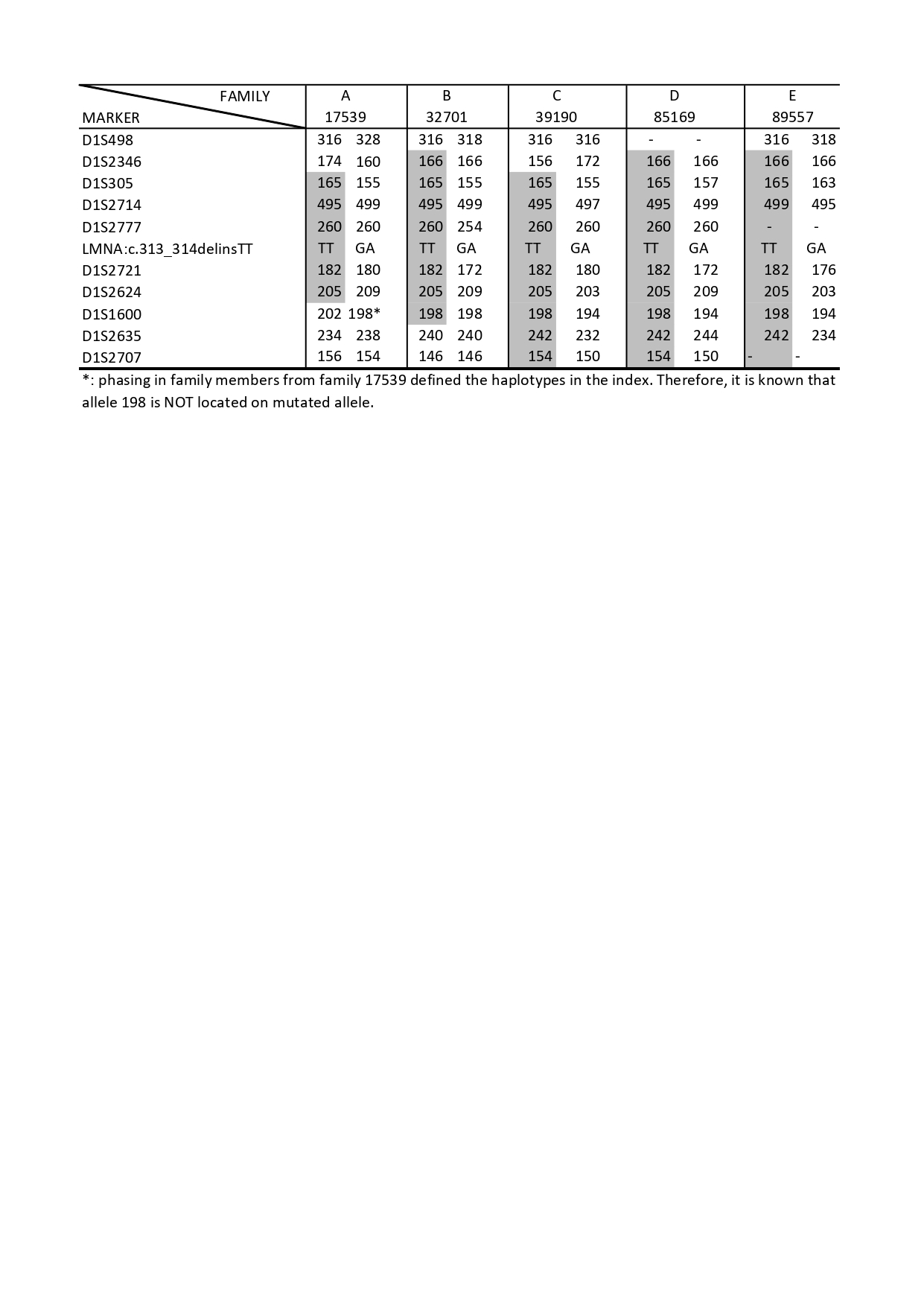 |
| --- |
| **Supplemental Figure 2. The haplotype associated with the c.313_314delinsTT variant in five families**. Schematic representation of the haplotypes on chromosome 1, locus 1q21.3-23.1. The disease haplotype is shaded. |

| 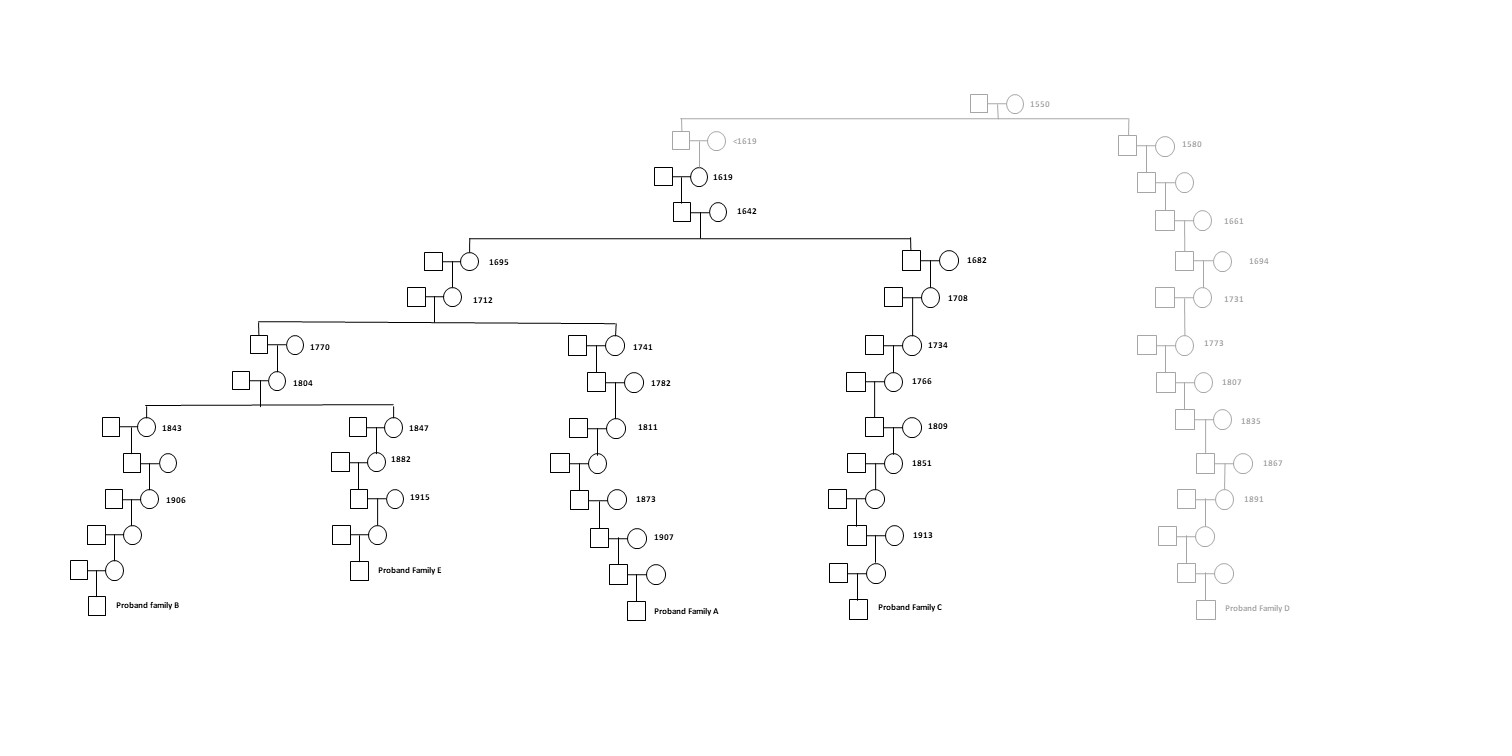 |
| --- |
| **Supplemental Figure 3. Genealogical pedigree of families carrying the *LMNA* p.(Glu105Leu) variant.** Squares represent males, circles represent females. Probands are indicated in the figure. Families A, B, C, and E were genealogically linked 10 generations ago to a village at the Dutch-German border. Family D was linked to the other families 3 generations above, although final genealogical evidence is still lacking (shown in grey). The estimated origin of the variant is between 643 and 656 years ago (25.7 to 26.2 generations, assuming 25 years per generation). |

| 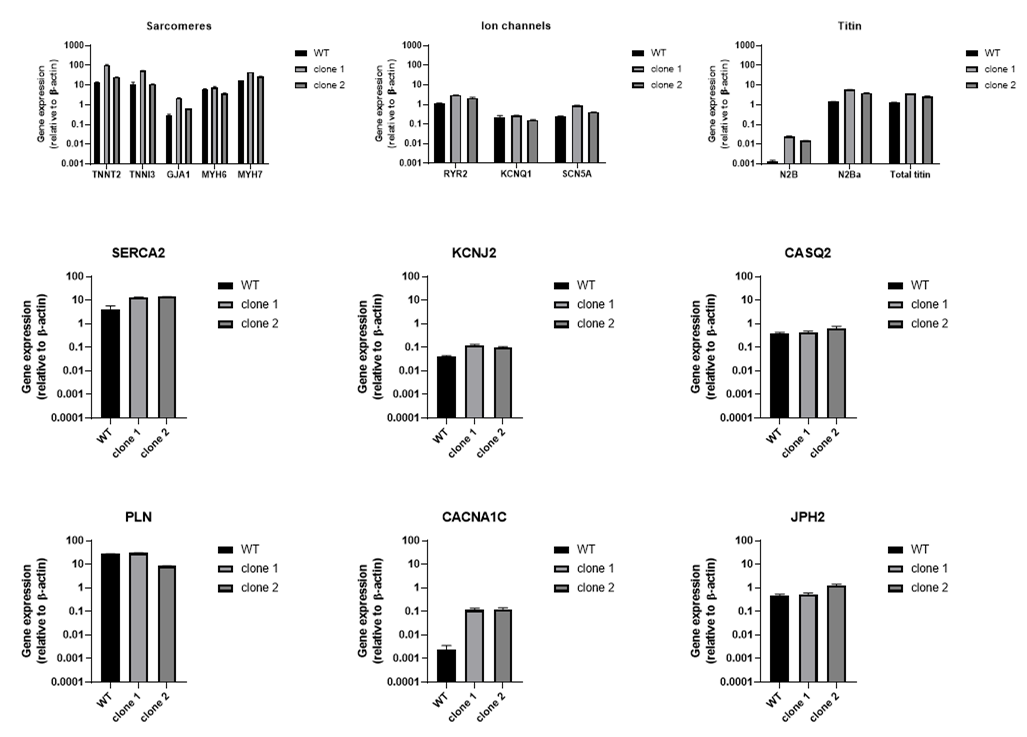 |
| --- |
| **Supplemental Figure 4. Gene expression analysis of *LMNA* p.(Glu105Leu) and wild-type iPSC-CMs. Bar graphs show the relative expression levels of mature cardiomyocyte-related genes in the *LMNA* p.(Glu105Leu) variant and wild-type iPSC-CMs.** The analyzed genes include sarcomere proteins (*TNNT2, TNNI3, GJA1, MYH6, MYH7*), ion channels (*RYR2, KCNQ1,* *SCN5A*), titin (*N2B, N2BA*) and calcium handling-related proteins (*SERCA2, KCNJ2, CASQ2, PLN, CACNA1C, JPH2*). Analysis suggests distinct but overlapping maturation profiles between *LMNA* p.(Glu105Leu) and wild-type iPSC-CMs. |

| **Wild-type** | |
| --- | --- |
| 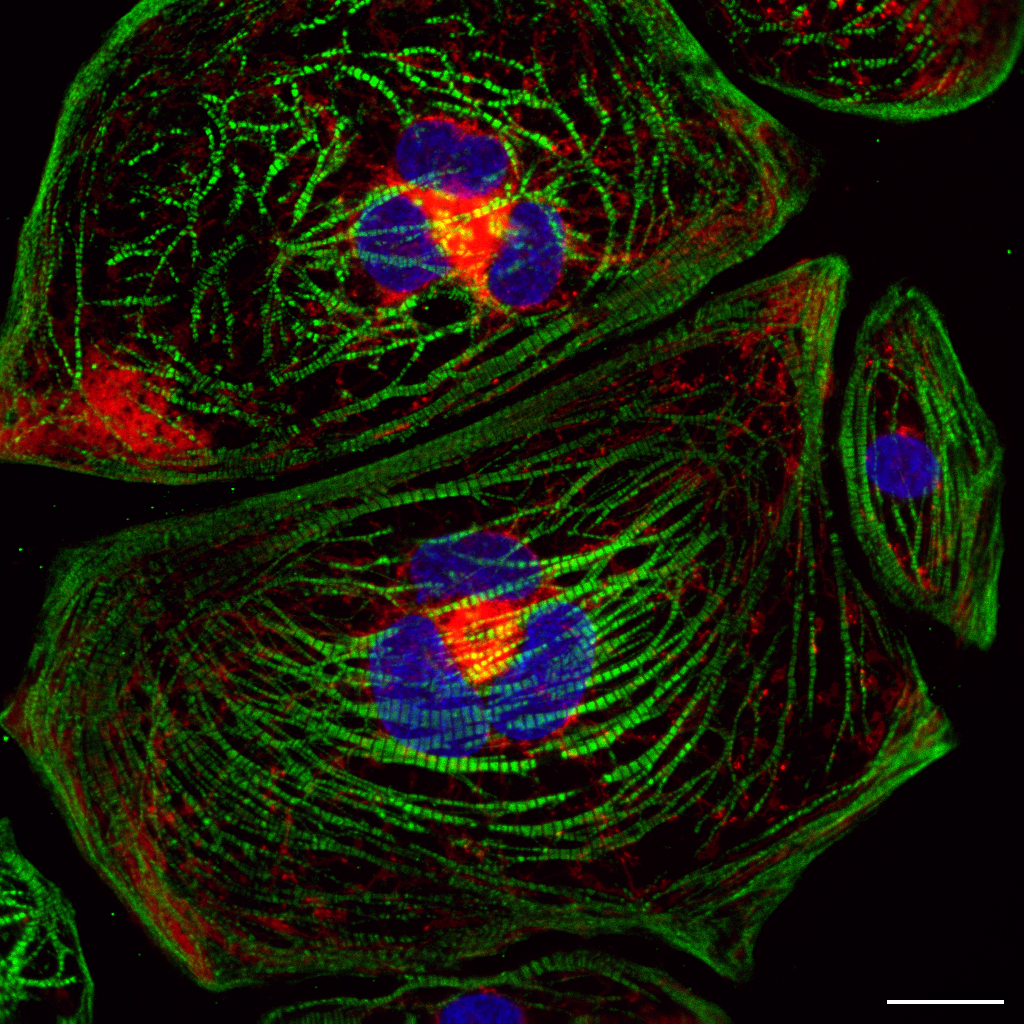 | **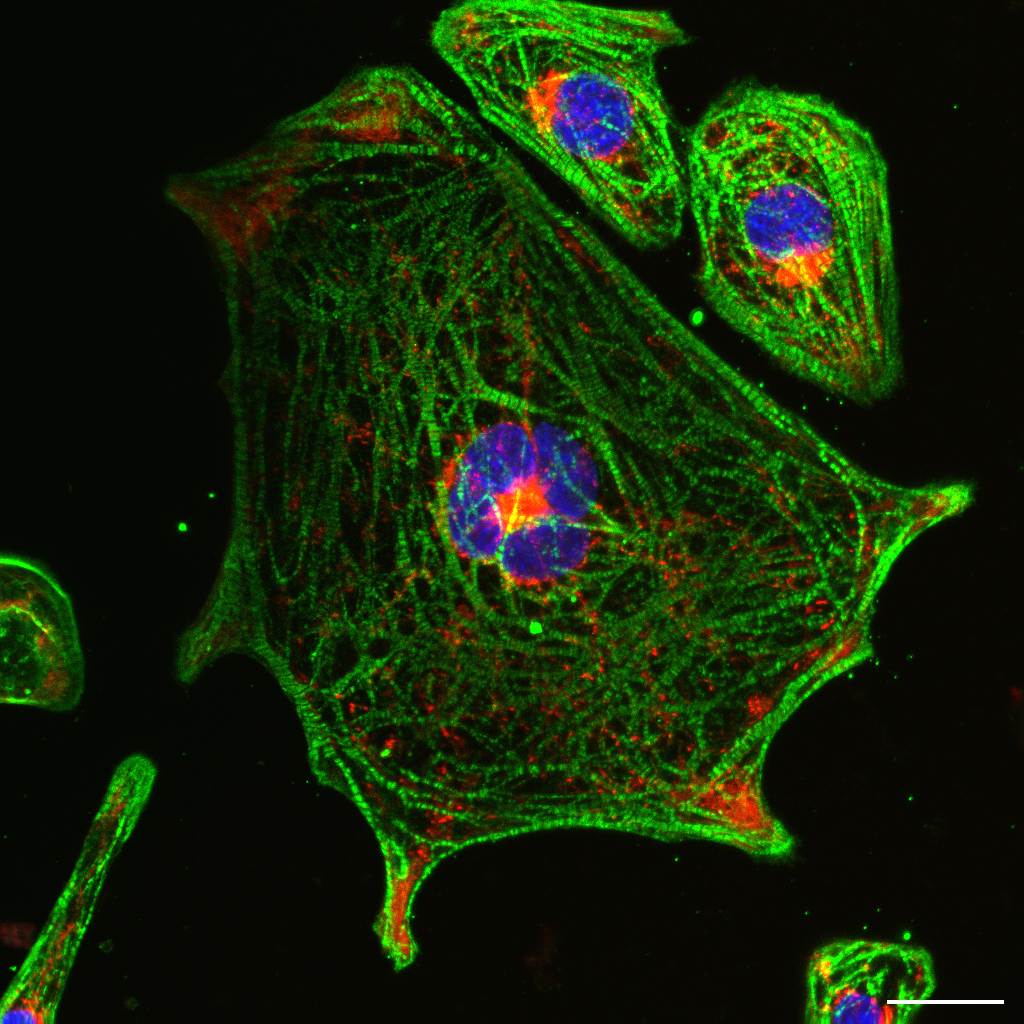** |
| **Clone 1** | |
| 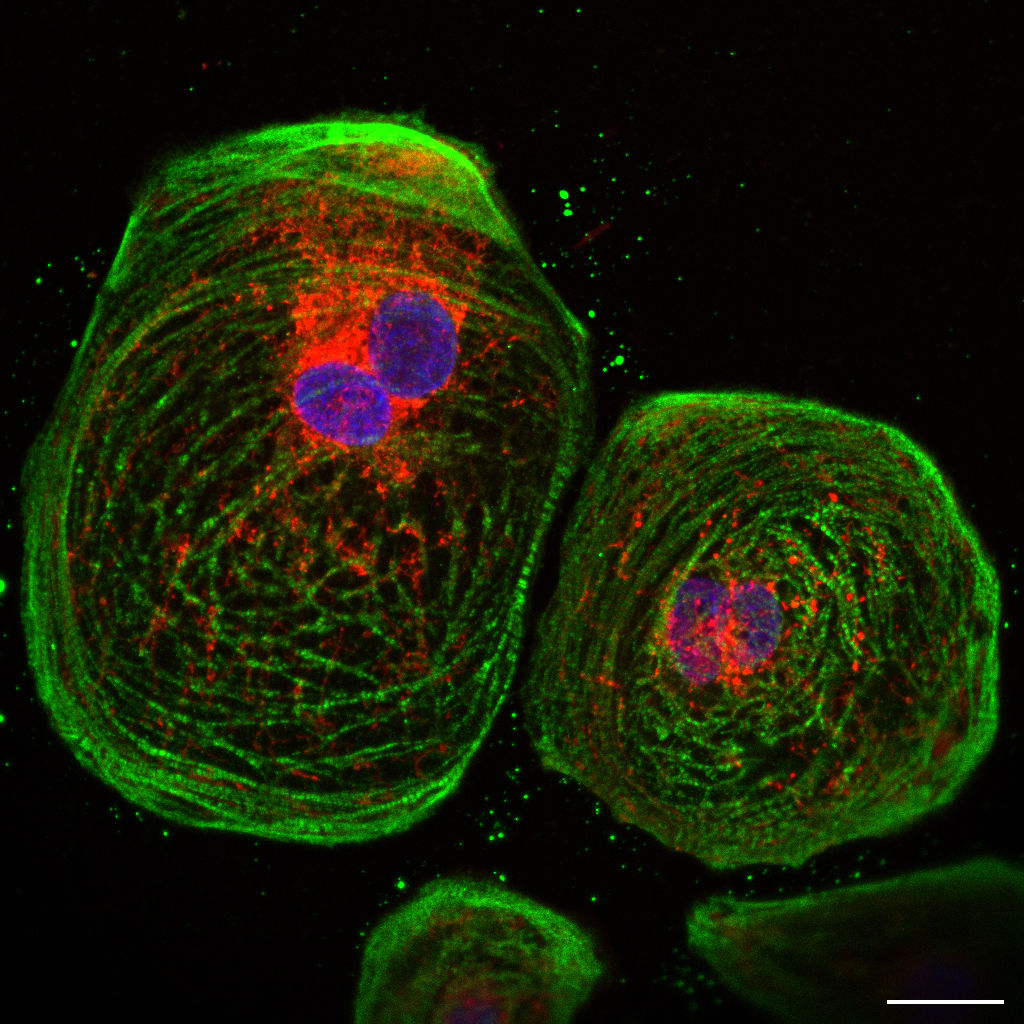 | **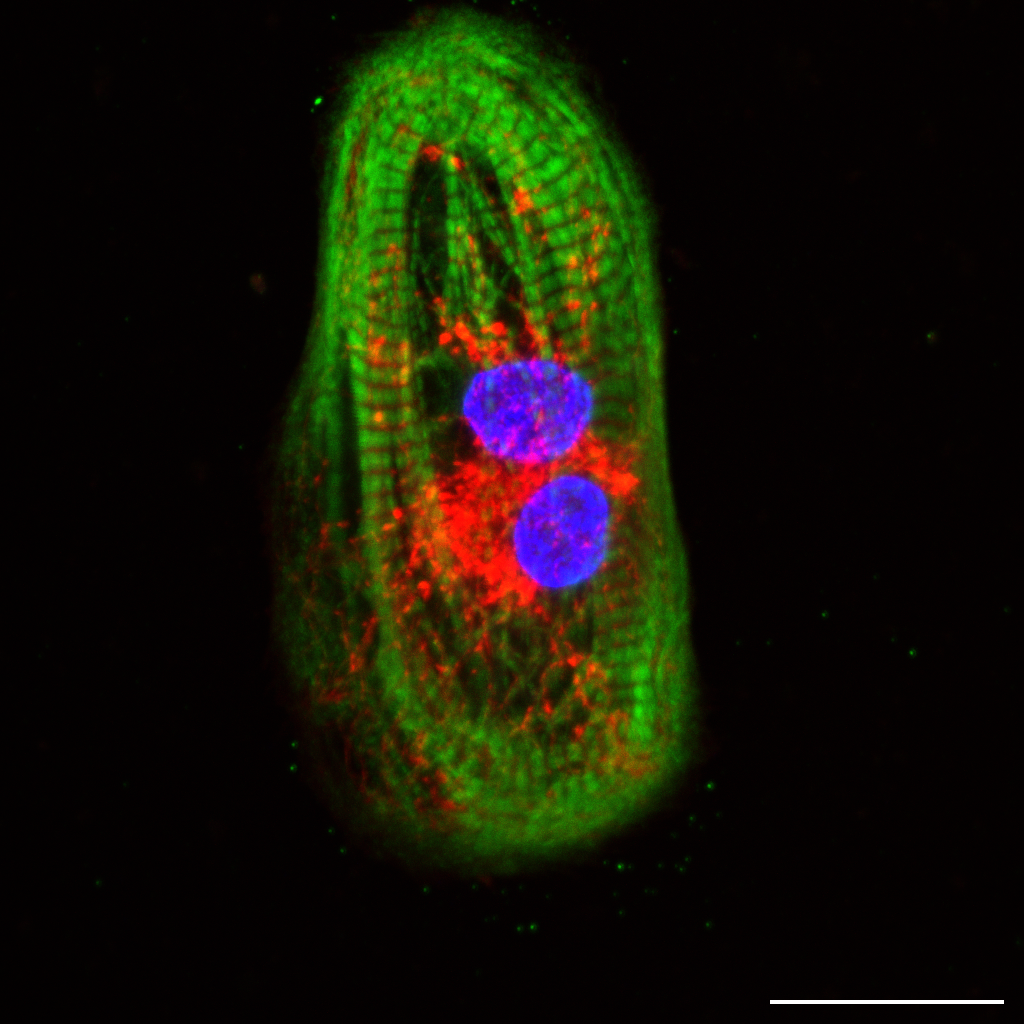** |
| **Clone** 2 | |
| 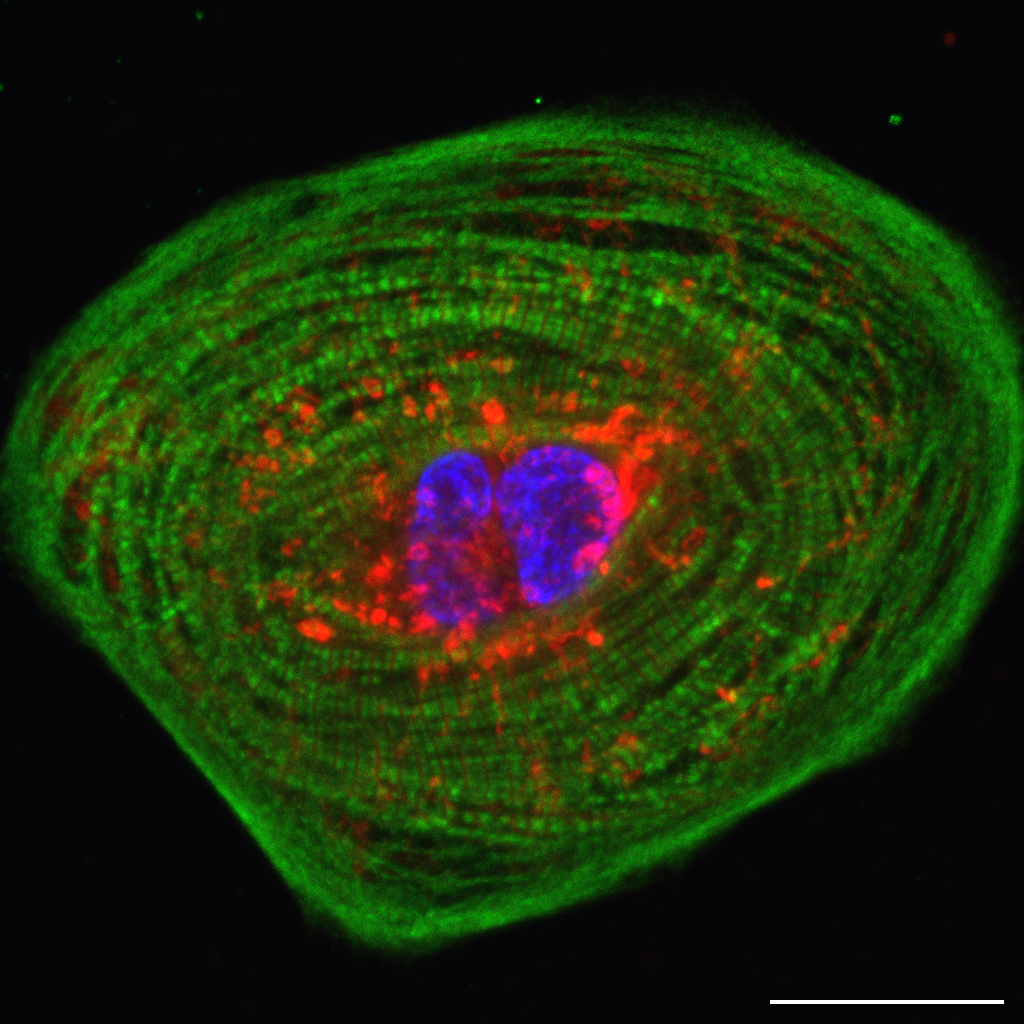 | **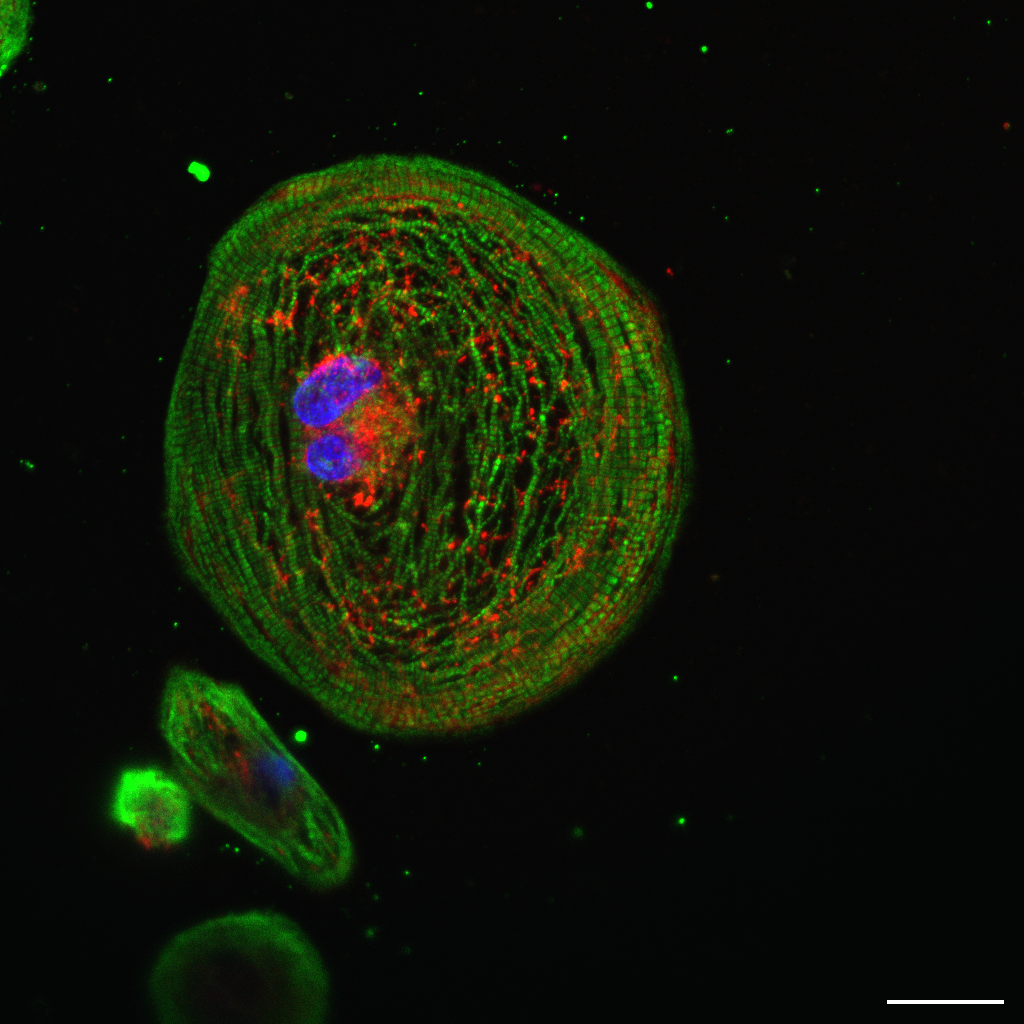** |
| **Supplemental Figure 6. Mitotracker Staining of *LMNA* p.(Glu105Leu) iPSC-CMs.** Representative images of Mitotracker staining in wild-type and *LMNA* p.(Glu105Leu) iPSC-CMs. Mitotracker staining reveals altered mitochondrial distribution in *LMNA* p.(Glu105Leu) iPSC-CMs compared to control cells, supporting the findings from TEM analysis (**Figure 5G**). In wild-type iPSC-CMs the mitochondria are more located around the nucleus, whereas in the LMNA p.(Glu105Leu) iPSC-CMs they are more dispersed throughout the cardiomyocyte. Scale bar = 20 µm. | |

| *LMNA p.(Glu105Leu) IPSC-CMs*  Clone 1 Clone 2  **M** 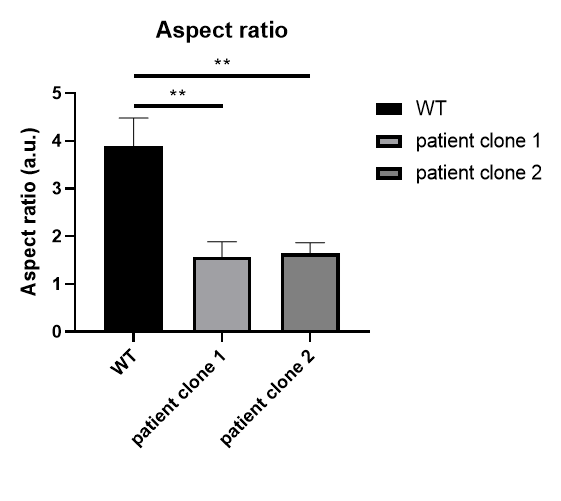 **N** 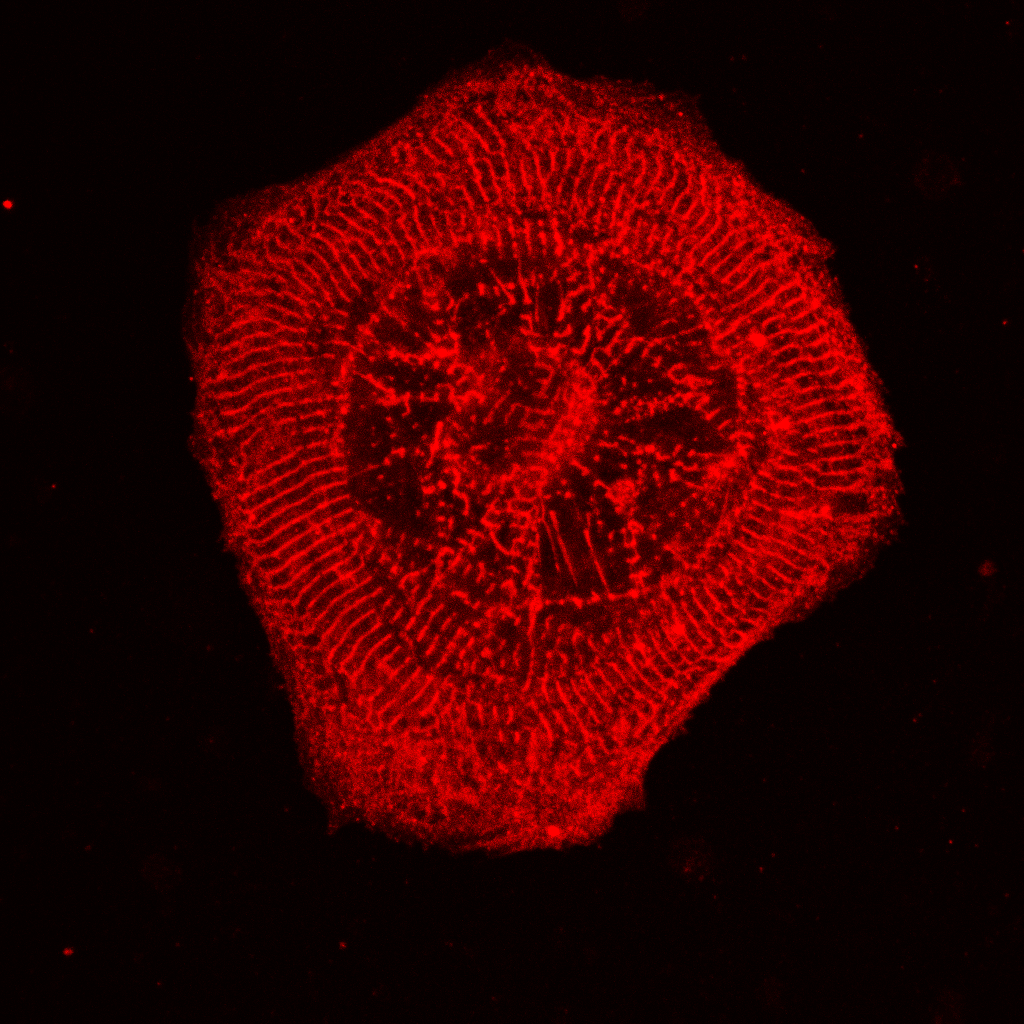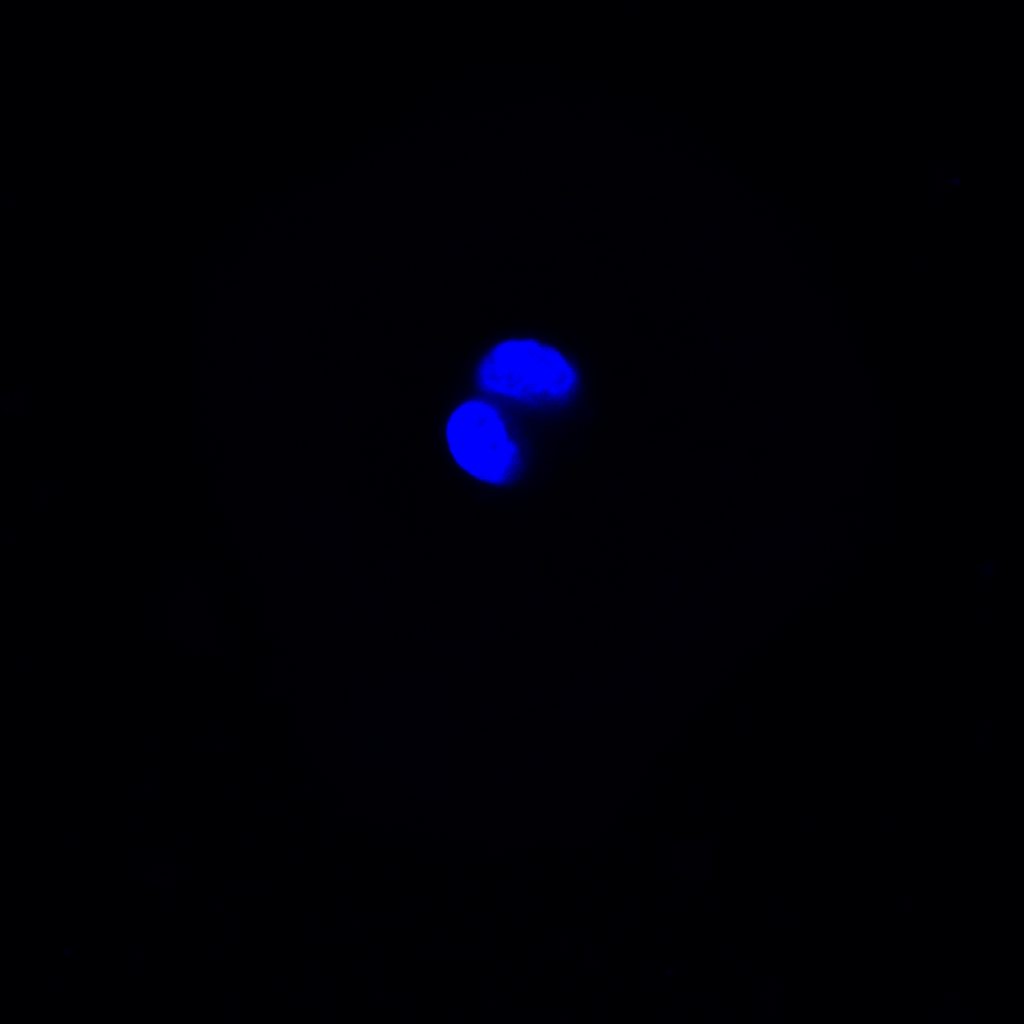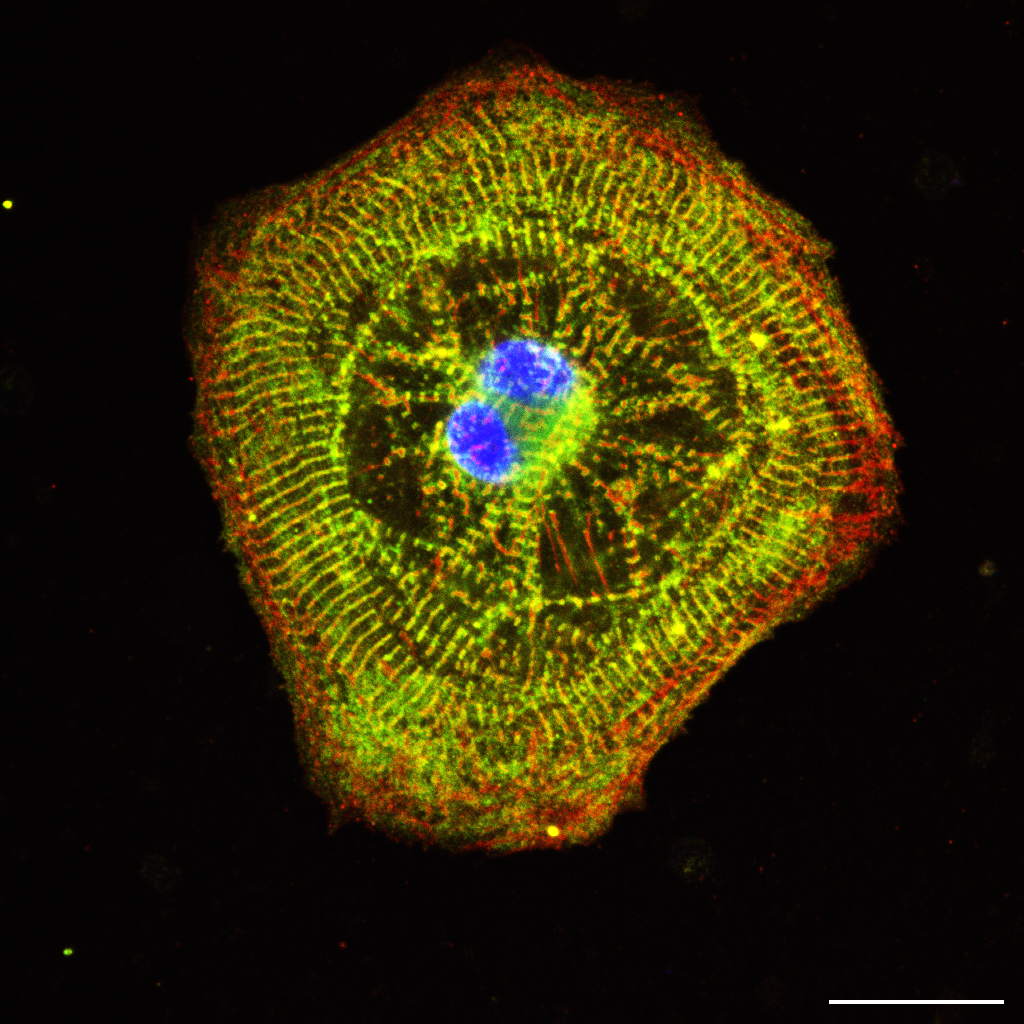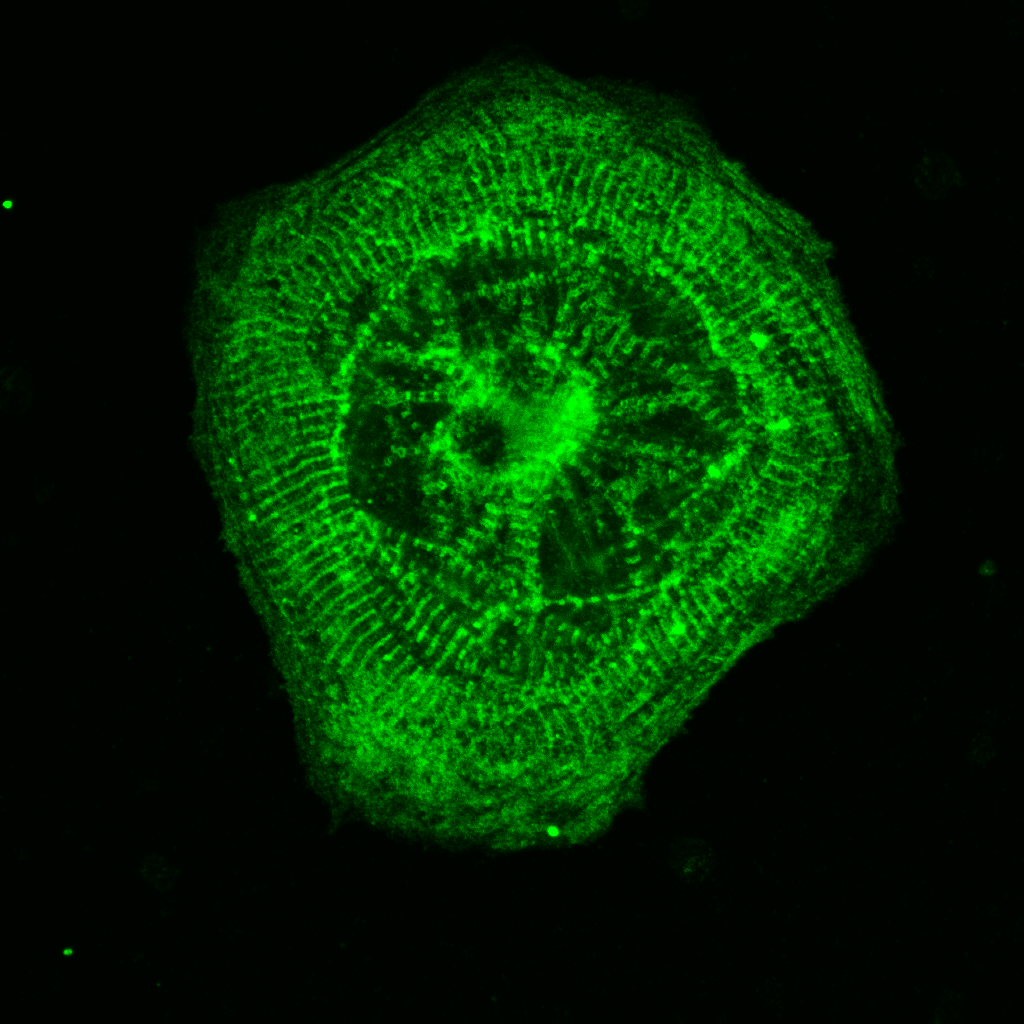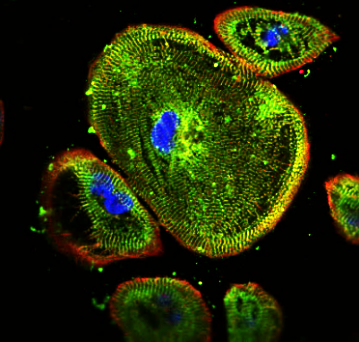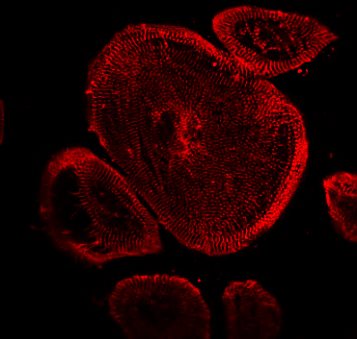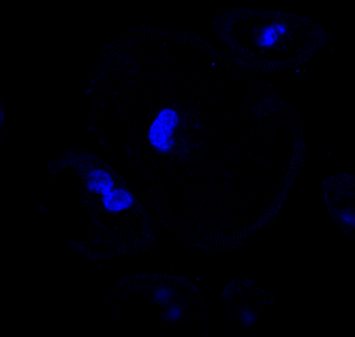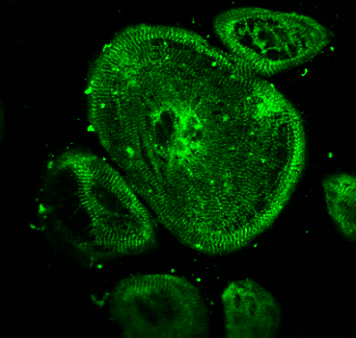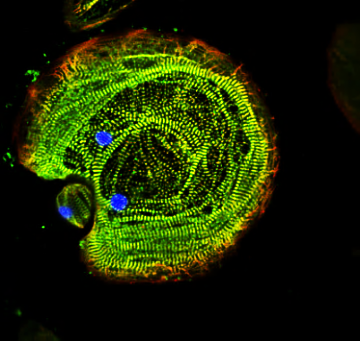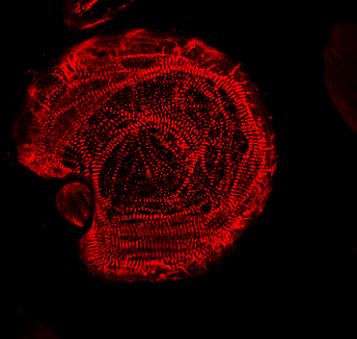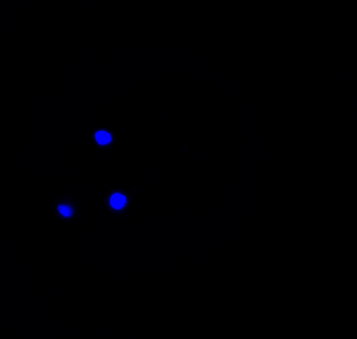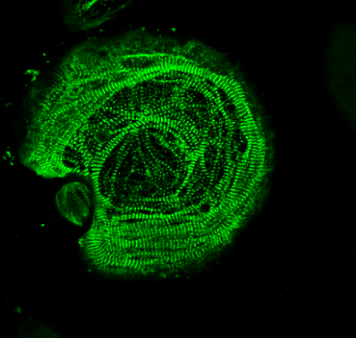 WT iPSC-CMs  TTN  α-actinin  Hoechst  Merged  **A**  **B**  **C**  **D**  **E**  **F**  **G**  **H**  **I**  **J**  **K**  **L** |
| --- |
| **Supplemental Figure 7. Immunohistochemical analysis and quantitative assessment of sarcomeric organization in wild-type and *LMNA* p.(Glu105Leu) iPSC-CMs.** Representative immunofluorescence images show (**A-C**) titin (TTN, green), (**D-F**) $\alpha$-actinin (red), and (**G-I**) nuclei (Hoechst, blue) staining in wild-type and *LMNA* p.(Glu105Leu) iPSC-CMs. (**J-L**) Merged images show the spatial relationship between sarcomeric proteins and nuclear morphology. These images and analysis demonstrate the sarcomeric disarray in *LMNA* p.(Glu105Leu) iPSC-CMs. Scale bar: 20 µm. (M) Quantitative analysis presents the sarcomere dispersion, indicating the variability sarcomere orientation. (N) Aspect ratio of the iPSC-CMs, reflecting the cell shape. Data were represented as mean ± SEM and plotted on Graphpad Prism (n = 3). Statistical significance was assessed using one-way ANOVA and unpaired t-test. *p<0.05, **p<0.01, ***p<0.001. |

| **Wild-type** | | |
| --- | --- | --- |
| **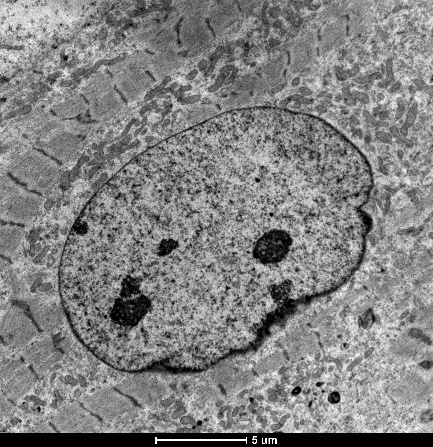** | **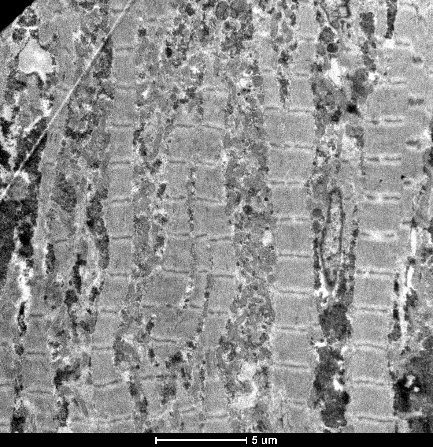** | 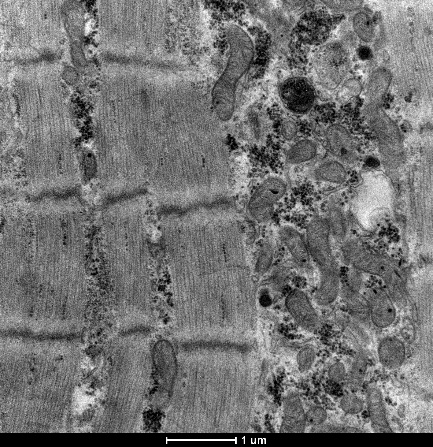 |
| **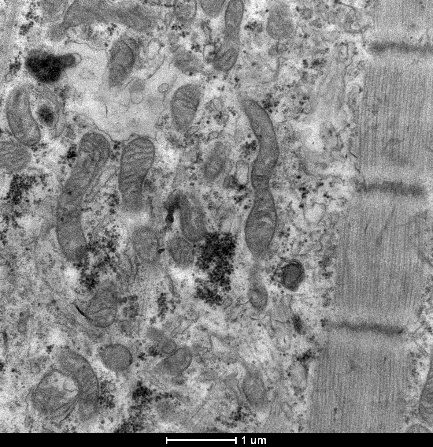** | **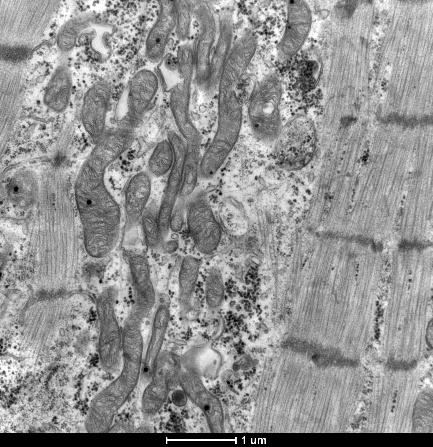** | 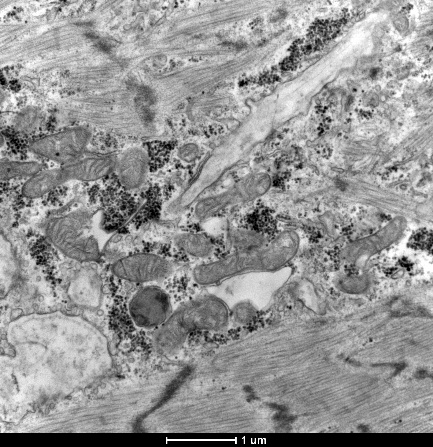 |
| **Clone 1** | | |
| **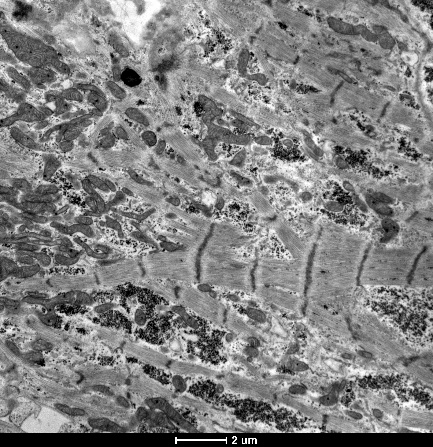** | **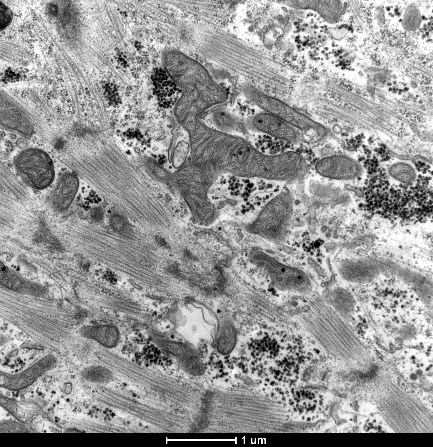** | 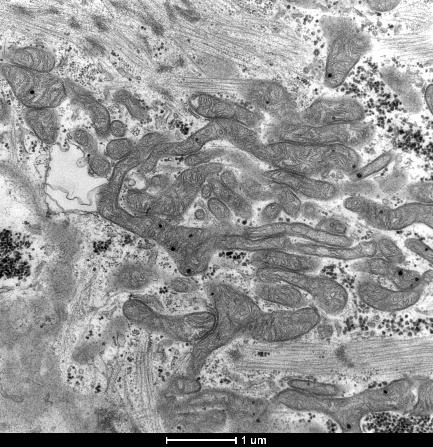 |
| **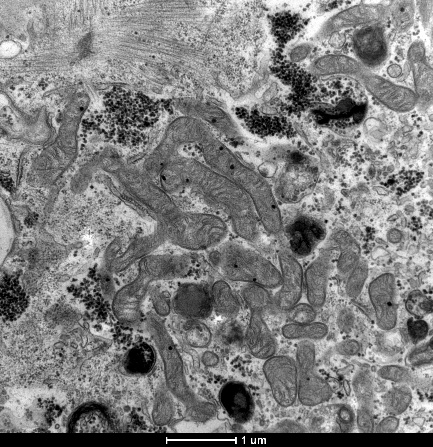** | **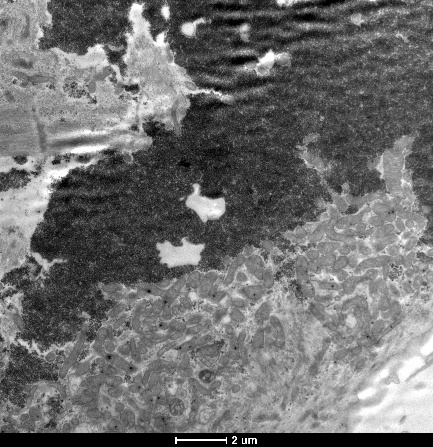** | 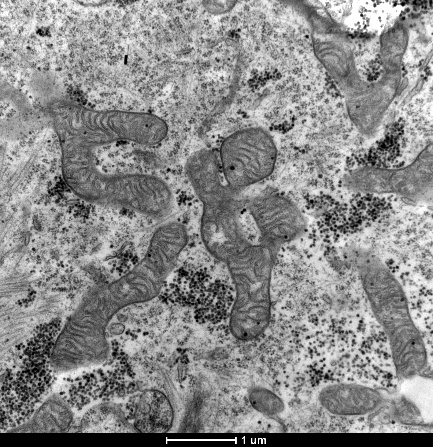 |
| **Clone 2** | | |
| 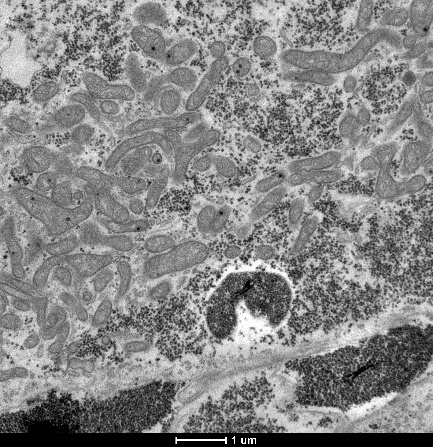 | 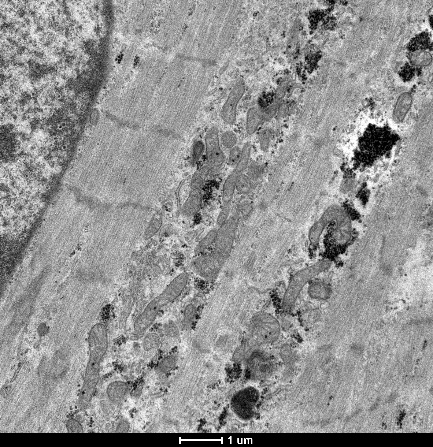 | 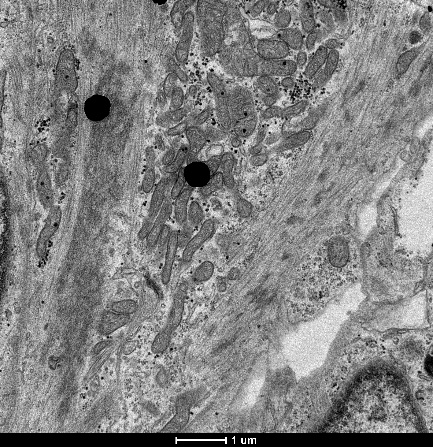 |
| 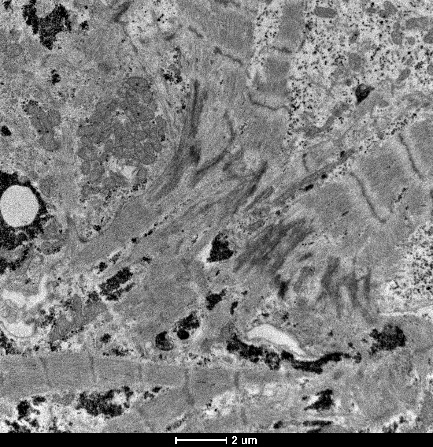 | 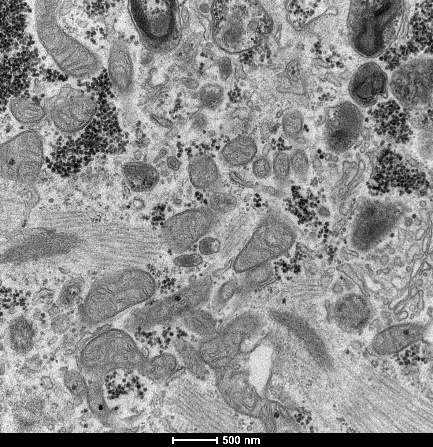 | 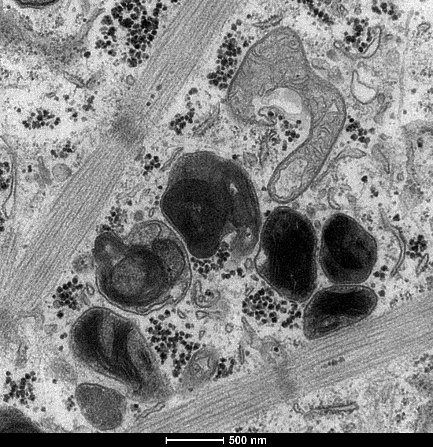 |
| **Supplemental Figure 8. Additional TEM Images of *LMNA* p.(Glu105Leu) iPSC-CMs**. Additional TEM images of *LMNA* p.(Glu105Leu) iPSC-CMs showing further examples of structural abnormalities, including sarcomeric disorganization, mitochondrial alterations, and glycogen accumulation. These images complement the findings presented in **Figure 5F-H**, providing additional qualitative evidence of the cellular phenotypes associated with the *LMNA* p.(Glu105Leu) variant. | | |
